# Distinct pre-initiation steps in human mitochondrial translation

**DOI:** 10.1038/s41467-020-16503-2

**Published:** 2020-06-10

**Authors:** Anas Khawaja, Yuzuru Itoh, Cristina Remes, Henrik Spåhr, Olessya Yukhnovets, Henning Höfig, Alexey Amunts, Joanna Rorbach

**Affiliations:** 1 Department of Medical Biochemistry and Biophysics, Division of Molecular Metabolism, Karolinska Institutet, Biomedicum, 171 65 Solna, Sweden; 2 Max Planck Institute Biology of Ageing - Karolinska Institutet Laboratory, Karolinska Institutet, Stockholm, Sweden; 3 Science for Life Laboratory, Department of Biochemistry and Biophysics, Stockholm University, 17165 Solna, Sweden; 4 Department of Mitochondrial Biology, Max-Planck-Institute for Biology of Ageing, Joseph-Stelzmann-Str. 9b, 50931 Cologne, Germany; 5 RWTH Aachen, I. Physikalisches Institut (IA), Aachen, Germany; 6 Forschungszentrum Jülich, Institute of Complex Systems ICS-5, Jülich, Germany

**Keywords:** Cryoelectron microscopy, Mitochondria, Ribosome

## Abstract

Translation initiation in human mitochondria relies upon specialized mitoribosomes and initiation factors, mtIF2 and mtIF3, which have diverged from their bacterial counterparts. Here we report two distinct mitochondrial pre-initiation assembly steps involving those factors. Single-particle cryo-EM revealed that in the first step, interactions between mitochondria-specific protein mS37 and mtIF3 keep the small mitoribosomal subunit in a conformation favorable for a subsequent accommodation of mtIF2 in the second step. Combination with fluorescence cross-correlation spectroscopy analyses suggests that mtIF3 promotes complex assembly without mRNA or initiator tRNA binding, where exclusion is achieved by the N-terminal and C-terminal domains of mtIF3. Finally, the association of large mitoribosomal subunit is required for initiator tRNA and leaderless mRNA recruitment to form a stable initiation complex. These data reveal fundamental aspects of mammalian protein synthesis that are specific to mitochondria.

## Abstract (web summary)

Translation within mitochondria relies upon specialized mitoribosomes and initiation factors. Here the authors use Cryo-EM and single molecule approaches to describe the early steps leading to translation initiation in human mitochondria, and the roles of mitochondria-specific ribosomal proteins and initiation factors mtIF2 and mtIF3.

## Introduction

The genome of human mitochondria encodes for thirteen proteins that require specialized mitoribosomes for their synthesis. The specialization of the mitoribosomes is illustrated by the reduction of rRNA and addition of specific proteins, for many of which the functions are not known^1,2^. The process of protein synthesis starts with translation initiation. The canonical bacterial translation begins with the initiation factors IF1, IF2, IF3, and initiator tRNA that assemble on the small ribosomal subunit (SSU) to recognize the mRNA start codon, followed by the joining of the large subunit (LSU). The Shine–Dalgarno (SD) sequence is used to recruit mRNA to SSU through the anti-SD of the rRNA. However, in human mitochondria the mRNAs and the mitoribosome do not have the respective SD and anti-SD sequences. In addition, IF1 is missing compared to the bacterial system, whereas the GTPase mtIF2 and mtIF3 have specific extensions. Mitochondrial mRNAs are also different as most of them do not possess 5′ leader sequences, further highlighting special requirements for translation initiation.

A complete mitochondrial translation initiation complex with the joint mitoribosomal subunits has been reconstituted from bovine components^3^, and mtIF3 has been identified as a crucial component for initiation in mouse^4^. However, how the initiation complex is formed and which structural elements of the mitoribosome, initiation factors, and the interplay between them lead to the full assembly is not known. To investigate how translation initiation starts in human mitochondria, we analyze here the early involved complexes of the small mitoribosomal subunit using a combination of cryo-EM, fluorescence cross-correlation spectroscopy, and single-molecule fluorescence techniques. Our data reveal two defined steps that lead to the mitochondrial translation initiation, termed mitochondrial preinitiation steps 1 and 2 (mtPIC-1, mtPIC-2), which explains how mitochondria-specific extensions of initiation factors interact with mitoribosomal proteins to govern the mechanism of translation.

## Results

### Structure determination of mitochondrial preinitiation translation complexes

**Fig. 1:**
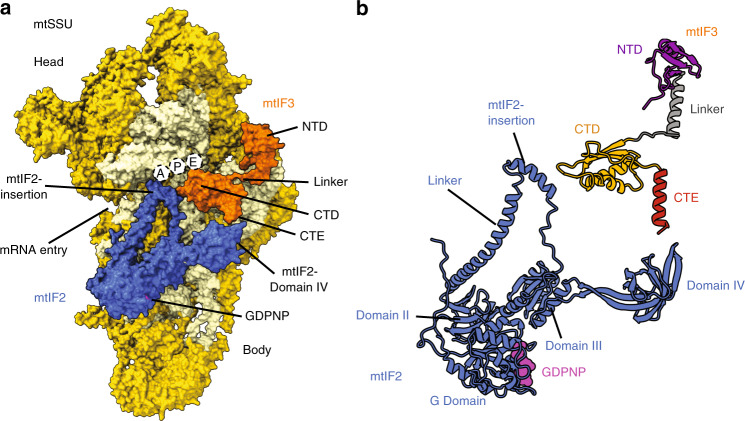
Structure of the human mitochondrial preinitiation translation complex (mtPIC-2). **a** Surface representation of the mtSSU with mtIF3 (orange) and mtIF2 (blue). Empty tRNA-binding sites and mRNA channel are indicated. The binding of mtIF2 is accomplished due to mtIF3 NTD restricting the mtSSU head movement. **b** Relative positions of mtIF3 colored by domains (NTD purple, linker dark gray, CTD orange, CTE red) and mtIF2.

We first purified the mtIF3-bound mitoribosomal SSU (mtSSU) by immunoprecipitation from HEK293 cells overexpressing 3xFLAG-tagged mtIF3. In bacteria, the preinitiation complex can be formed by adding IF2 to the SSU-IF3 complex in the presence of a nonhydrolyzable GTP analogue (GDPNP), formylmethionyl-tRNA^Met^_i_ (fMet-tRNA^Met^_i_), and mRNA^5^. Therefore, we incubated the pulled-down mtSSU-mtIF3 with purified recombinant human mtIF2 in the presence of GDPNP, *Escherichia coli* fMet-tRNA^Met^_i_, and leaderless mRNA (MT-CO2). To determine the structure of the complex, a cryo-EM density map was calculated from a subset of 552,920 particles and signal subtraction was applied using a mask for the entire complex except the mtIF3-binding site, followed by 3D classification using the mask for the mtIF3-binding site (Supplementary Fig. 1). Subsequently, particles containing mtIF3 were further classified with signal subtraction on mtIF2, which resulted in two maps at 3.0 Å and 3.1 Å resolution, containing mtIF3 and mtIF2-mtIF3, respectively (Supplementary Figs. 2–5, Supplementary Tables 1, 2). No fMet-tRNA^Met^_i_ or mRNA was detected on the mtSSU, suggesting that the identified arrangements are the most stable and likely represent states prior to the binding of tRNA and mRNA, namely mitochondrial preinitiation (mtPIC) (Fig. 1).

### The conformational rigidity of mtIF3 preserves vacant mtPIC

**Fig. 2:**
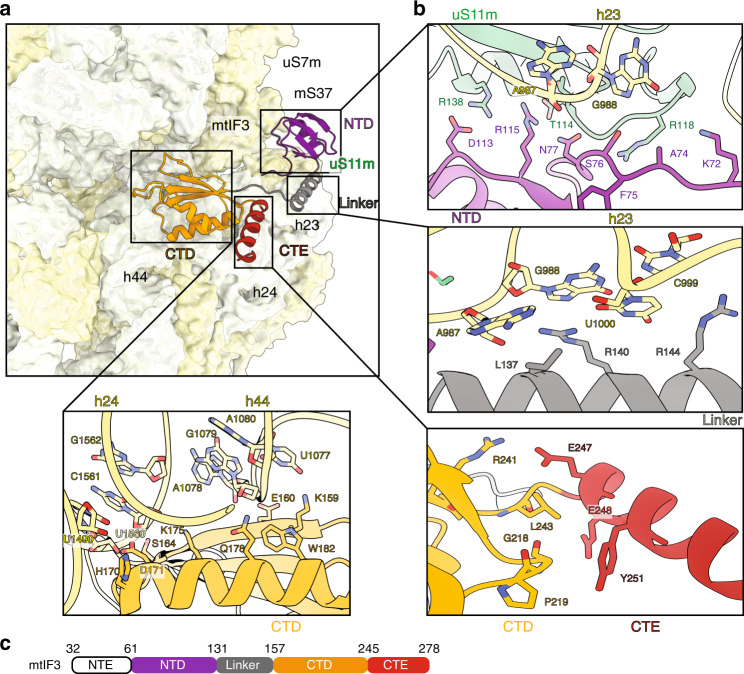
Multiple interactions of mtIF3 with the mtSSU. **a** Domain organization of mtIF3 on mtSSU with mitochondria-specific CTE (red) positioned outward from mtSSU. **b** Zoom-in panels for each of the mtIF3 domains featuring interactions with mtSSU. **c** Schematic representation of the mtIF3 with the corresponding color-code. NTE is disordered in the structure.

In bacteria, the conventional description of the translation preinitiation pathway implies large-scale changes in IF3 that define its distinct role during the process, including accommodation of the fMet-tRNA^Met^_i_ into the P site for start codon recognition^5^. In mitochondria, mtIF3 adopts a more extended topology that includes N- and C-terminal domains (NTD, CTD) flanked by mitochondria-specific extensions (NTE, CTE) and joined by a helical linker (Fig. 2). In our mtSSU-mtIF3 (mtPIC-1) structure, the CTD is bound to h24 (1077–1080) and h44 (1480, 1560–1562), where the rRNA sequence differs from bacteria (Fig. 2, Supplementary Table 3). This mode of binding overlaps with two conserved inter-subunit rRNA bridges B2a and B2b, blocking the premature association of the mitoribosomal LSU (mtLSU). In addition, the helical linker would interfere with H68 of the mtLSU. The NTD residues of mtIF3 (S76, N77, D113, and R115) also form interactions with h23 and uS11m (T114, R118, and R138) close to uS7m and mS37 (Fig. 2a, Supplementary Table 3), while in bacteria no substantial interactions with IF3-NTD have been reported^5^ (Supplementary Fig. 6a). The NTD residues of mtIF3 that interact with the mtSSU are highly conserved amongst vertebrates, but not in bacteria (Supplementary Fig. 6c).

The position of CTD also overlaps with the fMet-tRNA^Met^_i_ binding site on mtSSU, which is similar in bacteria where IF3-CTD needs to be relocated on SSU to accommodate fMet-tRNA^Met^_i_^5^. Prior to this, bacterial IF3-NTD moves away from the platform to about 36 Å from its original position, to bind the elbow of fMet-tRNA^Met^_i_. This relocation is unlikely in mitochondria, due to the multiple contacts between mtIF3 and mtSSU. Moreover, while in bacteria the surface of the NTD of IF3 that interacts with fMet-tRNA^Met^_i_ is positively charged, allowing RNA-protein interaction, mtIF3-NTD has a negative electrostatic potential (Supplementary Fig. 6b) and the residues that interact with the tRNA elbow in bacteria are not conserved in mtIF3 (Supplementary Fig. 6c). This implies that mtIF3-NTD cannot interact efficiently with tRNA. Importantly, the residues responsible for tRNA discrimination in bacteria^6,7^ are not conserved in mtIF3 (Supplementary Fig. 6c).

These data suggest an extensive network of contacts between mtIF3 and mtSSU that synergistically stabilize the association. Such mode of binding provides structural constraints that limit the range of conformational flexibility of mtIF3 on the mitoribosome in the preinitiation state, preventing it from sampling different positions. Therefore, mtIF3 appears to be more rigid on the mitoribosome, which is in contrast to bacterial species, where IF3 flexibility is essential for tRNA binding along the translation preinitiation pathway.

The observed conformation of the mitochondria-specific CTE of mtIF3 can also contribute to preserving vacant mtPIC. The CTE is an α-helix facing away from the mtSSU (Supplementary Fig. 7a) and its orientation is stabilized by specific interactions with the CTD (Fig. 2b). The CTE occupies the binding site of the acceptor stem of the fMet-tRNA^Met^_i_, illustrated by superimposition with the complete initiation complex containing fMet-tRNA^Met^_i_, which shows mutual exclusivity with mtIF3 (Supplementary Fig. 7b). Therefore, mtIF3 has to leave to allow the complete initiation complex to fully assemble. The comparison of the mtSSU-mtIF3 interactions with the recently reconstituted complex of bovine mtSSU and human mtIF3^8^ shows that most of them have not been identified before (detailed analysis is given in Supplementary Fig. 8 and Supplementary Table 3).

**Fig. 3:**
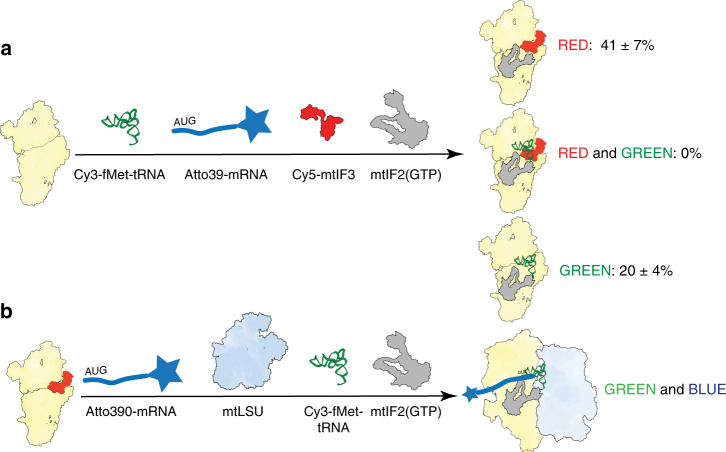
Binding of mtIF3 and fMet-tRNA^fMet^_i_ to the mtSSU is mutually exclusive. **a** Two-color fluorescence cross-correlation spectroscopy analysis of the initiation complex components. The mtSSU was incubated with an excess of Cy3-fMet-tRNA^fMet^_i_, Cy5-mtIF3, mtIF2(GTP), and Atto390-mRNA, then purified by a sucrose gradient. Products may contain unlabeled mtIF2. **b** The functionality of mtPIC was validated by the ability to form a complete initiation complex when incubated with an excess of mtLSU, Cy3-fMet-tRNA^fMet^_i_, Atto390-mRNA, and unlabeled mtIF2 (GTP). After purification of the monosome, detection of both Cy3 and Atto390 fluorophores confirmed binding of mRNA and tRNA. Source data are provided as a Source Data file.

To further investigate the formation of the translation initiation complex, we used in vitro fluorescence cross-correlation spectroscopy with fMet-tRNA^Met^_i_, mtIF3, and MT-CO2 mRNA labeled with spectrally distinct fluorophores, in the presence of unlabeled mtIF2. The analysis showed that mtIF3 and fMet-tRNA^Met^_i_ are not found on the same mtSSU particles (Fig. 3a, Supplementary Fig. 9). The functionality of the mtPIC was then analyzed by its ability to form a complete initiation complex when mixed with the mtLSU in the presence of mtIF2 (GTP), Cy3-labeled fMet-tRNA^Met^_i_ and mt-mRNA labeled at the 3′ end with an Atto390 fluorophore (Fig. 3b). Correct formation of the complete initiation complex was confirmed by fluorescence emission of both the Atto390 and Cy3 fluorophores, revealing that both the mRNA and fMet-tRNA^Met^_i_ are bound. Notably, no binding of mt-mRNA to the mtSSU was observed in our experimental conditions (Supplementary Table 4).

### Mitoribosomal protein mS37 restricts the head swiveling for the accommodation of mtIF2

In bacteria, binding of IF3, IF1, and mRNA to the SSU stabilizes a swiveled conformation of the SSU head with respect to the body that consequently allows IF2 to bind and advance to the next step in the initiation process^5,9^. However, mitochondria lack IF1, and as our structure shows, no mRNA is detected in mtPIC-1. Therefore, to reveal the mechanism for stabilization of the mtSSU head in mtPIC-1, we performed a computational analysis of the head movement.

**Fig. 4:**
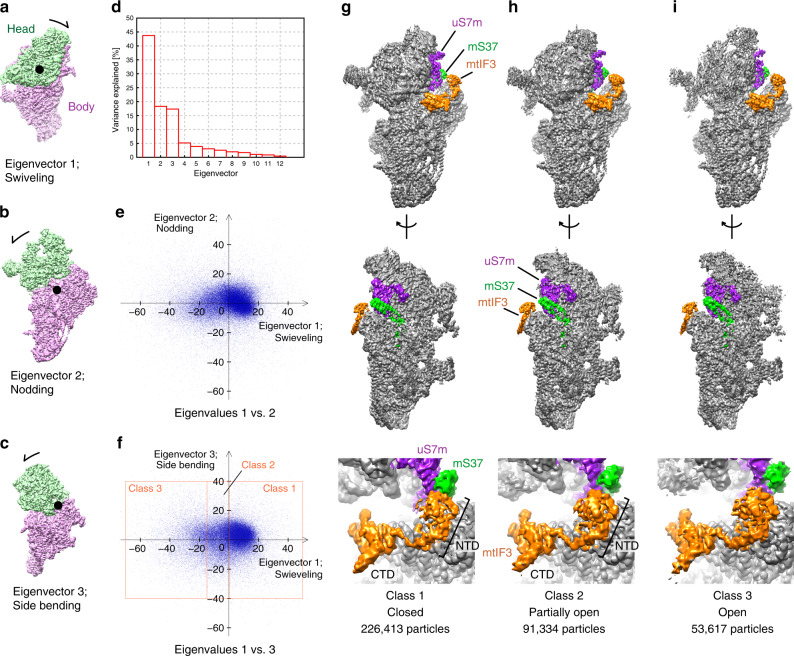
Principal component analysis of the mtSSU head motion suggests a function for the mitoribosomal protein mS37 in restricting the swiveling. **a**–**c** The relative orientation of the head to the body is described by 12 eigenvectors, representing translation and rotation in each particle. The eigenvectors 1, 2, and 3 were found to mainly contribute to the head motion, as indicated. **d** Histogram of the variances explained by the 12 eigenvectors. **e**, **f** The 1st to 3rd eigenvalues of randomly chosen particles represented by dots are plotted against each other, showing non-gaussian distribution for the swiveling motion. The particles are classified based on the values of eigenvector 1 (rectangles). **g**, **h** Comparison between the reconstructions of the classified particles. The closed conformation (Class 1) exhibits weaker density for the mtIF3-NTD interacting with mS37 and uS7m, compared to the other conformations with higher particle numbers.

In the first step, the 3D classification showed similar but distinct head positions, indicating a continuous movement. To better distinguish between the states and rationalize the motion, we subsequently performed 3D multibody with a principal component analysis of the relative orientations of the head and body for all the particle images^10^ (Fig. 4). The relative orientations are mainly described by three eigenvectors and only the vector that corresponds to the head swiveling shows a non-Gaussian distribution, suggesting that this motion is the most prominent in mtPIC-1 (Fig. 4d–f). Comparison between maps based on head swiveling establishes a correlation between the position of mS37 and the presence of mtIF3-NTD. In particular, when mS37 is in its closest proximity to the mtIF3-binding site (closed state), the density for mtIF3-NTD is weaker (Fig. 4g–h), suggesting their incompatibility.

The mS37 is a mitochondria-specific 13.5 kDa protein, with a coiled-coil-helix domain^11^, peripherally bound at the mRNA channel exit^1^. Its motion around the mtSSU vertical axis is driven by the rotation of the internal rRNA h28, which forms the neck. The h28 is most compressed in the closed state, as can be seen through the movement of equivalent atoms in different states (Supplementary Fig. 10). A careful inspection of the density in this region reveals an alternative conformation of the h28-associated region of uS7m (β-hairpin motif I156-P173 and the C-terminus N233-W282) that represents an even more compressed conformation of h28 (Supplementary Fig. 10). Since a more closed head conformation would result in a clash with the mtIF3, it suggests that the functional role of mS37 is to restrict the degrees of freedom of the mtSSU head swiveling in mtPIC-1. Taken together, our structural information suggests that the interplay between mS37 and mtIF3 is the mechanism that keeps the mtSSU head a favorable position for the consequent accommodation of mtIF2 (Supplementary Fig. 11).

### The conformational rearrangement of mtIF2 is required for translation initiation progress

**Fig. 5:**
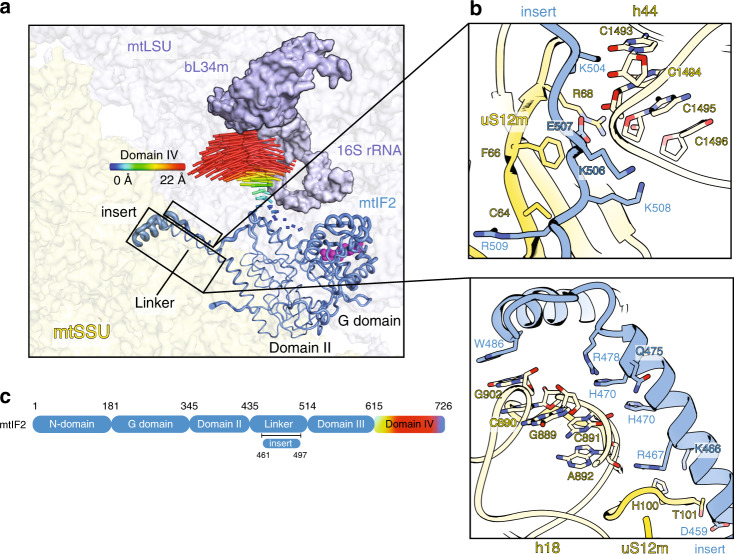
Contacts between mtIF2 and mtSSU in mtPIC-2, and conformational changes upon mtLSU association. **a** Comparison of mtIF2 binding in mtPIC-2 with the complete initiation complex (PDB ID: 6GAW). Upon association of the mtLSU (blue), required for complete initiation complex, the mtIF2-domain IV moves towards the mtSSU. The conformational change is represented by the difference vectors for each C_α_ atom of domain IV. For the other domains, the flexibility is represented through ribbon thickness according to B-factor. **b** Zoom-in panels showing contacts of the mtIF2-specific insert and linker domain with the mtSSU. **c** Schematic representation of the mtIF2 with the corresponding color-code to the conformational changes.

The factor mtIF2 is a multi-domain GTPase that comprises four conserved domains and a mitochondria-specific insertion of 37 amino acids between its helical and linker regions (Fig. 5). In our mtSSU-mtIF2-mtIF3 (mtPIC-2) structure, mtIF2 adopts a similar conformation to its bacterial counterpart^5,12^, but with some important differences. The α-helical mtIF2 insertion spans the same binding pocket as bacterial IF1, blocking the A-site, as previously suggested^13,14^ (Fig. 1). The insert establishes contacts mostly through hydrogen bonding with h18 (889–892) and uS12m (Fig. 5, Supplementary Table 5). The linker comprising the loop region (K504-R509) connected to domain III interacts with h44 (1493–1496) and uS12m (Fig. 5b, Supplementary Table 5). These specific interactions of mtIF2 with mtSSU can affect the conformational space of the mtSSU head, which is different from mtPIC-1 and bacterial counterparts, as revealed by the 3D multibody analysis (Supplementary Fig. 12). Thus, the data suggest that the role of mtIF2 in mtPIC-2 is to stabilize the assembly and prepare it for the following step.

During the next step of the initiation pathway, the mtLSU is accommodated. To understand the structural rearrangements required for this event to occur, we compared the structures of mtPIC-2 and the complete initiation monosome complex^3^. Our analyses revealed three major features: (i) the mtIF2 globular domain IV undergoes a profound conformational change, reflected in 22Å movement for R613, whereas the C-terminus remains anchored (Fig. 5a); (ii) h44 is rearranged, losing interaction with the mtIF2 linker (Supplementary Fig. 13 and 14, Supplementary Table 5); (iii) the G-domain of mtIF2 is found in two different GTPase states, namely preactive in mtPIC-2 and active in the complete initiation complex. The G-domain-bound GDPNP is found in close proximity to the mtIF2-domain III and the C-terminus, and the transition between mtPIC-2 and the complete initiation complex involves a switch of the catalytic and conserved H238 (Supplementary Fig. 15). Particularly, in mtPIC-2, H238 is rotated away from the γ-phosphate of GDPNP, representing the preactive conformation, whereas in the complete complex it primarily adopts an inward facing conformation activating GTP hydrolysis. Moreover, the region encompassing the P-loop and switch I display subtle rearrangements, particularly V189 and I213, which form the hydrophobic gate and appear important for the GTPase activity (Supplementary Fig. 15). The dynamics of mtIF2-domain IV between the two initiation stages causes a subtle reordering of its C-terminus, notably of F727, which lies adjacent to the catalytic H238 (Supplementary Fig. 15). The correlation between the mtLSU incorporation, the conformational change of h44 and mtIF2, and GTPase activation suggests that the flexibility of domain IV reported here is crucial for the formation of the complete initiation complex.

### Leaderless mt-mtRNA forms a stable complex with the monosome during translation initiation

**Fig. 6:**
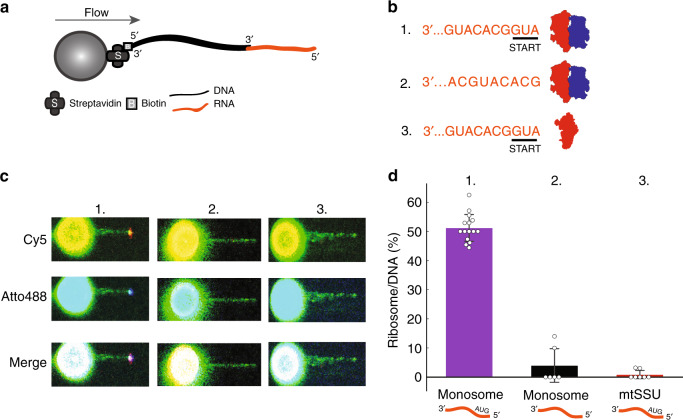
Binding of leaderless mt-mRNA to the monosome. **a** An optical tweezers setup to study mitochondrial translation initiation. RNA construct containing the first 51 nucleotides of MT-CO2 mRNA was ligated to biotinylated lambda DNA, allowing the 5′-end accessible for mitoribosome binding. **b** In order to monitor both mitoribosomal subunits, mtLSU and mtSSU were labeled with Cy5 and Atto488, respectively, and initiation reactions were made by incubating either double-labeled monosome (b, 1.) or Cy5-labeled mtSSU (b, 3.) with the DNA/RNA hybrid, mtIF3, mtIF2 (GTP), and fMet-tRNA^Met^_i_. An RNA construct without start codon was used as a negative control (b, 2.). **c** Representative images showing binding of the mitoribosomal subunits tested by simultaneous dual-color confocal imaging. **d** Quantification of the binding events. The number of detected fluorescently labeled species were normalized to the number of trapped imaged DNA. Bars represent mean ± SD. Source data are provided as a Source Data file.

In bacteria, binding of the canonical mRNA to the SSU, which involves base pairing between a SD and anti-SD sequence upstream of the start codon, is a prerequisite for subunits joining and formation of the complete initiation complex. Notably, neither in our structural studies nor fluorescence analysis was leaderless mt-mRNA detected on the mtSSU. Instead, mt-mRNA was only found in the complete initiation complex (Fig. 3b). To further investigate the binding of leaderless mRNA to the mitoribosome, initiation reactions were made by incubating MT-CO2 mRNA hybridized to biotinylated lambda DNA (Fig. 6a) with either double-labeled monosome (labeled with Cy5 at the mtSSU and with Atto488 at the mtLSU) or Cy5-labeled mtSSU (Fig. 6b). Binding of the mitoribosomal subunits to mRNA was examined by simultaneous dual-color imaging using C-Trap™ optical tweezers combined with confocal microscopy. In these experimental conditions, we observed binding of the leaderless mRNA construct (but not mRNA without a start codon) to the monosome. As observed by cryo-EM, no stable binding to the mtSSU was detected (Fig. 6c, d). This result is in-line with several studies showing that also bacterial leaderless mRNAs have a much higher affinity for monosome than SSU^15,16^.

## Discussion

**Fig. 7:**
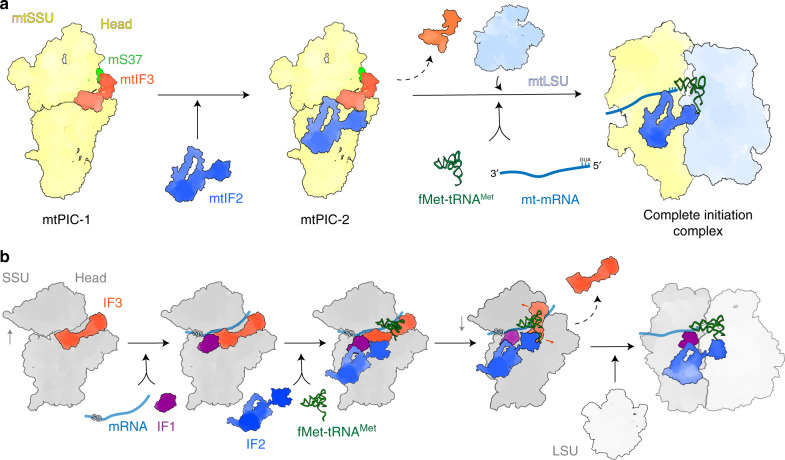
Model of mitochondrial translation initiation and comparison with the bacterial system. **a** Translation initiation in mammalian mitochondria. The various structures are summarized in the order that represents a possible initiation pathway in which mtIF2 binding precedes mtLSU and tRNA binding. In this pathway, mtIF3 and mS37 stabilize the mtSSU head for the accommodation of mtIF2. Joining of the mtLSU may result in the conformational change of mtIF2 and GTPase activation that leads to tRNA and mRNA accommodation. Alternatively, transient binding of both mRNA and tRNA (not detected with our techniques) to the mtSSU after mtIF3 departure precede recruitment of the mtLSU. **b** Translation initiation in bacteria. Binding of canonical mRNA, fMet-tRNA^Met^_i_ and initiator factors precede subunit joining and formation of the complete initiation complex.

In the present study, we applied cryo-EM and fluorescent analysis to reveal mitochondria-specific states leading to translation initiation in human cells. The data provide insights in to the role of the mitoribosomal-specific protein mS37 in stabilizing the mtSSU head in the presence of mtIF3, which is tightly bound to the mtSSU and prevents accommodation of the initiator tRNA. This state is defined as mtPIC-1. Subsequent accommodation of mtIF2 with preactivated GTPase results in the intermediate state, mtPIC2, that allows binding of the mtLSU, replacement of mtIF3 with initiator tRNA and accommodation of the mitochondrial leaderless mRNA, which leads to the formation of a complete elongation-competent initiation complex (Fig. 7).

Although we cannot explicitly state that the identified mtPIC-1 and mtPIC-2 are exclusive and rule out the possibility that other short-lived intermediate remained undetected, the stable association of mtIF2 and mtIF3 with the mtSSU, as well as mt-mRNA and mt-tRNA with the monosome, suggests that the visualized events are the most prevalent in the process of mitochondrial translation initiation (Fig. 7). Taken together, our results provide the snapshots of the non-canonical assembly of the translation initiation machinery in mitochondria that coevolved together with the distinct mt-mRNAs, mitoribosomal-specific proteins, and extensions of the initiation factors.

## Methods

### Generation of a mammalian cell line expressing mtIF3

The Flp-In T-Rex human embryonic kidney 293T (HEK293T) cell line (Invitrogen), which allows for the generation of stable doxycycline-inducible expression of transgenes by FLP recombinase-mediated integration, was used to express the human mitochondrial IF3 (mtIF3). HEK293T cells were grown in DMEM containing 10% tetracycline-free FCS (Clonetech) and supplemented with 100 μg/ml Zeocin (Invitrogen) and 15 μg/ml Blasticidin S (Invivogen, San Diego, CA). The pcDNA5/FRT/TO vector harbouring mtIF3 cDNA with its C-terminus carrying FLAG-tag preceded by an HRV 3 C cleavage sequence (PreScission Site): Leu-Glu-Val-Leu-Phi-Gln↓Gly-Pro and the pOG44 vector, harboring the FLP recombinase, were co-transfected with Lipofectamine3000 (Thermo Fisher), according to the manufacturer’s instructions. Transfected cells were selected adding 15 μg/ml Blasticidin S and 100 µg/ml Hygromycin B to culture media. Approximately 2 to 3 weeks post transfection, positive colonies appeared and single colonies were picked and expanded. Protein expression was induced adding doxycycline (Sigma–Aldrich) to a final concentration of 50 ng/ml to culture media 48 h prior experimental analysis.

### Preparation of the mtPIC

Cells overexpressing mtIF3 were collected, resuspended in an ice-cold hypotonic buffer (0.6 M mannitol, 100 mM Tris-HCl pH 7.5, 10 mM ethylenediaminetetraacetic acid (EDTA), 0.05% bovine serum albumin (BSA)) and ruptured using dounce-homogenizers. The intact mitochondria were isolated by differential centrifugation: the crude mitochondria were loaded on to the sucrose gradient (1.0 M and 1.5 M sucrose, 20 mM Tris-HCl pH 7.5, 1 mM EDTA) and centrifuged for 1 h at 25,000 rpm. (SW41 Ti rotor, Beckman Coulter). The band formed by the mitochondria between 1 M and 1.5 M sucrose was collected and resuspended in 10 mM Tris-HCl pH 7.5 in 1:1 ratio. After centrifugation, the purified mitochondrial pellet was resuspended in mitochondrial freezing buffer (200 mM trehalose, 10 mM Tris-HCl pH 7.5, 10 mM KCl, 0.1% BSA, 1 mM EDTA), flash-frozen and stored at −80 °C.

The mitoribosome bound FLAG-tagged mtIF3 was purified by immunoprecipitation. The purified mitochondria were lysed by incubating at 4 °C for 20 min in the lysis buffer (25 mM HEPES-KOH pH 7.5, 5.0 mM Mg(OAc)_2_, 100 mM KCl, 2% (v/v) Triton X-100, 0.2 mM dithiothreitol (DTT), 1X cOmplete EDTA-free protease inhibitor cocktail (Roche), 40 U/µl RNase inhibitor (Invitrogen). The lysate was centrifuged at 5000 × *g* for 5 min at 4 °C and the supernatant was added to ANTI-FLAG M2 Affinity Gel (Sigma–Aldrich), equilibrated with the wash buffer (25 mM HEPES-KOH pH 7.5, 5.0 mM Mg(OAc)_2_, 100 mM KCl, 0.05% β-DDM). After 3 h incubation at 4 °C, the gel was washed with the wash buffer and mtIF3-bound complexes were eluted by additional incubation of 2 h with the PreScission protease (GE Healthcare) (2 U/µl). Prior to the addition, the original storage buffer of the protease was replaced by the wash buffer with 2.0 mM DTT by buffer exchange using the concentrator Vivaspin MWCO 30,000 PES (Sartorisu).

### Preparation of mtIF2, mtIF3, MT-CO2 mRNA, *E. coli* fMet-tRNA^Met^_i_

A codon-optimized (Genscript) DNA construct corresponding to the mature form of human mtIF2 (amino acids 38–727) or human mtIF3 (amino acids 32–278) was cloned into a pET-24b vector (Novagen). Both constructs were expressed in Rosetta 2 cells (EMD chemicals) at 25 °C for 16 h in Magic Media (Thermo Fisher Scientific). After lysis, the proteins were purified over a His-Select Ni^2+^ resin (Sigma–Aldrich) and dialyzed against H-0.2 (25 mM Tris-HCl pH 7.4, 0.5 mM EDTA, 10% glycerol, 1 mM DTT, 200 mM NaCl). Further purification was conducted over a HiLoad 16/60 Superdex 200 pg gel filtration column (GE Healthcare) in buffer H-0.2 lacking glycerol.

The first 30 or 51 nucleotides of mitochondrially encoded cytochrome C oxidase 2 (MT-CO2) mRNA was purchased from Sigma–Aldrich or Eurofins, respectively.

The unlabeled *E. coli*, fMet-tRNA^Met^_i_ was generously provided by Prof. Marina Rodnina, and the fMet-tRNA^Met^_i_ labeled at the dihydrouridine residues with Cy3 by Prof. Barry S. Cooperman^17^.

### Reconstitution of the mtPIC

The formation of the human mitochondrial mtPICs was achieved through initial purification of the mtSSU-bound mtIF3. The recombinant mtIF2 (1 µM) was preincubated with GDPNP (2.5 mM) to form mtIF2-GDPNP in the presence of buffer A (25 mM HEPES-KOH pH 7.5, 5.0 mM Mg(OAc)_2_, 100 mM KCl, 0.05% β-DDM) for 30 min at room temperature. The mtIF2-GDPNP mix was added to the mtIF3-bound mtSSU eluate (A_260_ = 7.9) together with the fMet-tRNA^Met^_i_ (1 µM) and MT-CO2 mRNA (1 µM) and incubated for another 30 min at room temperature. Subsequently, samples were directly applied to prepare the cryo-EM grids.

### Cryo-EM and image processing

Holy carbon grids (Quantifoil R2/2, 300 mesh) were covered with a thin carbon layer (~3 nm thickness) and glow-discharged with 20 mA for 30 s. The reconstituted sample (3 µl) was applied to the grids at 4 °C with 100% humidity. After 30 s incubation, the grids were blotted for 3.5 s and vitrified by plunge-freezing in liquid ethane using a Vitrobot™ Mark IV (Thermofisher). Images were collected on a Titan Krios electron microscope (Thermofisher) operated at 300 kV and equipped with a K2 Summit direct electron detector (Gatan). Micrographs were obtained from automated data collections (EPU software, Thermofisher) at ×165,000 magnification, yielding a pixel size of 0.83 Å. 4 s exposures yielded a total dose of ~30 e^−^/Å^2^ in 20 frames, with defocus values ranging from −0.25 to −5.0 μm. A total of 13,831 micrographs were recorded and kept. Movie frames were aligned and averaged by global and local motion corrections by the program MotionCor2^18^. Contrast transfer function (CTF) parameters were estimated by Gctf ^19^. Particles were picked by Gautomatch and 2D classified by RELION 3.0^10^. In the first round, particles were picked by Gaussian based picking, followed by reference-free 2D classification. Several representative 2D classes were used as references for the second-round picking. The picked particles were subjected to 2D classification to discard contaminants as well as mtLSU and monosome particles (Supplementary Fig. 1). The remaining particles underwent 3D refinement by RELION 3.0^10^ using EMDB-2880^3^ as a 3D reference, followed by 3D classification with local angular search to remove poorly aligned particles. Well-resolved classes were pooled and subjected to 3D refinement and CTF refinement by RELION 3.0.

For classifying on mtIF3, signal subtraction was performed by RELION 3.0 using a mask covering the entire complex except for mtIF3-binding site, followed by 3D classification without alignment using the mask covering only mtIF3-binding site. The particles with mtIF3 were further classified on mtIF2 by the same procedure as that for mtIF3, by using the corresponding masks. The P-site was also subjected to focus classification to detect any density of the initiator tRNA, which found out no detectable tRNA occupancy. The two classes, mtSSU-mtIF3 and mtSSU-mtIF3-mtIF3, underwent 3D refinement and postprocessing (Supplementary Table 1). Reported resolutions are based on gold-standard refinement, applying the 0.143 criterion on the FSC between reconstructed half-maps (Supplementary Fig. 2).

To improve the local resolution of mtSSU, four masks were prepared to cover the body core, the head core, tail, and mS39 and performed local-masked 3D refinement (Supplementary Figs. 3a and 4a). To improve the local resolution of the factors, local-masked 3D refinement was also performed using the masks covering the factors and the neighbouring regions (Supplementary Figs. 3b and 4b). Maps were sharpened and filtered by local resolution by using RELION 3.0.

### Model building and refinement

The models were manually built with *Coot*^20^. Ligands, metal ions, and modifications were placed based on the density. Hydrogens were generated to have better clash scores. Stereochemical refinement was performed using phenix.real_space_refine in the PHENIX suite^21^. The information on built proteins is in Supplementary Table 2. The final model was validated using MolProbity^22^. Refinement statistics are given in Supplementary Table 1. Figures were generated using PyMOL^23^, UCSF Chimera^24^, and UCSF ChimeraX^25^.

### 3D multibody analysis

3D multibody analysis was done by RELION 3.0^10^. To analyze the overall conformational change of mtSSU, two masks, head and body masks, were used. The head mask covers the head core and mS39, while the body mask covers the body core and tail as well as the factors (mtIF3 for mtPIC-1 and mtIF2 and mtIF3 for mtPIC-2). Eigenvalues of 100,000 randomly chosen particles are plotted each other for representation. Prior to classification, cleaning was done by removing outliers based on all of the 12 eigenvalues. After cleaning, mtPIC-1 and mtPIC-2 have 371,361 and 99,199 particles, respectively. Particles of mtPIC-1 are classified into three groups based on the eigenvector 1, while those of mtPIC-2 are classified into five groups based on the eigenvectors 1 and 2, as shown in Fig. 4f and Supplementary Fig. 12c.

### Purification of mitoribosomal subunits for in vitro experiments

Mitoribosomes were purified as previously described^26^ with minor modifications. Briefly, HEK293T cells were cultured in 3 L spinner flasks (Corning) in FreeStyle Expression Media (Gibco) until a density of 4 × 10^6^ cells/mL, washed with PBS and incubated in swelling buffer (25 mM HEPES-KOH pH 7.5, 100 mM KCl, 20 mM Mg(OAc)_2_, 2 mM DTT) for 20 min at 4 °C. The buffer was then supplemented with sucrose and mannitol to a final concentration of 70 mM and 210 mM, respectively, and the cells were disrupted in a glass homogenizer. The membranes were afterwards centrifuged at 1000 × *g* for 10 min, the supernatant was collected and centrifuged at 10,000 × *g* for 10 min. The pellet consisting of crude mitochondria was dissolved in mitochondria isolation buffer (25 mM HEPES-KOH pH 7.5, 100 mM KCl, 20 mM Mg(OAc)_2_, 70 mM sucrose, 210 mM mannitol, 2 mM DTT, supplemented with cOmplete protease inhibitor (Roche)) and treated with 10 U/mL DNase I for 20 min at 4 °C. The mitochondria were then treated with 0.2% digitonin in mitochondria isolation buffer for 5 min and washed two times with mitochondria isolation buffer. Lysis followed, by incubation in lysis buffer (25 mM HEPES-KOH pH 7.45, 100 mM KCl, 20 mM Mg(OAc)_2_, 2% Triton X-100, 2 mM DTT, supplemented with cOmplete protease inhibitor and RNase inhibitor) for 20 min at 4 °C. The membranes were pelleted by centrifugation at 23,500 rpm in a TLA-100.4 rotor for 20 min, and the mitochondrial lysate was then overlaid on top of a 10–30% sucrose gradient prepared in ribosome isolation buffer (25 mM HEPES/KOH pH 7.5, 100 mM KCl, 20 mM Mg(OAc)_2_, 2 mM DTT). The gradient was centrifuged at 21,000 rpm for 16 h in an SW41 Ti rotor, and later fractionated using a Piston Gradient Fractionator (Biocomp). The fractions corresponding to the monosomes were pulled and pelleted by centrifugation at 55,000 rpm in an SW60 rotor. To prepare mitoribosomal subunits, monsomes were incubated in dissociation buffer (20 mM HEPES-KOH pH 7.6, 300 mM KCl, 5 mM MgCl_2_, 1 mM DTT) for 3 h at 4 °C. The sample was then overlaid on top of a 10–30% sucrose gradient prepared in dissociation buffer and ultracentrifuged for 17 h at 21,000 rpm in an SW41 Ti rotor. The gradient was fractionated using a Piston Gradient Fractionator (Biocomp) and the fractions corresponding to mtSSU and mtLSU were pooled and centrifuged at 55,000 rpm in an SW60 rotor for 16 h. The resulting pellets were dissolved in initiation buffer (50 mM Tris-HCl, pH 7.6, 30 mM KCl, 10 mM MgCl_2_, 1 mM DTT, 0.1 mM spermine, 1 mM spermidine) and stored at −80 °C.

### Site-specific labeling of mtIF3

Labeling of mtIF3 was adapted from previously published protocol^27^, attaching the maleimide functionalized Cy5 fluorophore to the native Cysteine (Cys20). First, the DTT present in the storage buffer was removed by overnight dialysis in labeling buffer (50 mM Tris-HCl pH 7.1, 100 mM NH_4_Cl, 0.1 mM EDTA). Then, disulfide bonds were reduced by incubation in 10-time excess of Tris(2-carboxyethyl)phosphine hydrochloride (TCEP) for 10 min at 37 °C. The labeling reaction was made by incubation of 500 nM mtIF3 with 5-time excess of Cy5-maleimide for 3 h at room temperature. The excess of dye was removed by passing the reaction through two consecutive Zeba™ Spin Desalting Columns, 7 K MWCO. The labeled mtIF3 was then dialysed against storage buffer (20 mM Tris-HCl pH 7.1, 1 mM EDTA, 10% glycerol, 6 mM β-mercaptoethanol) and stored at −80 °C. The degree of labeling was determined by UV-VIS spectroscopy as 0.75 Cy5 per mtIF3.

### Formation of the preinitiation complex

Purified mtSSU (150 nM) were incubated with 750 nM mtIF2, 750 nM Cy5-labeled mtIF3, 750 nM Cy3-labeled fMet-tRNA^Met^_i_, and 5 mM GTP (in the presence or absence of Atto390-labeled mRNA) in initiation buffer for 15 min at 37 °C and 10 min on ice. The reaction was then overlaid on top of a 10–30% sucrose gradient in Initiation buffer and centrifuged in an SW41 Ti rotor at 21,000 rpm for 17 h. The gradient was afterwards fractionated using a Biocomp Piston Fractionator and the fractions containing mtSSU were pooled and concentrated using Vivaspin 500 centrifugal concentrators up to a volume of 50 µL.

### Formation of the initiation complex

To assemble the initiation complex, the preinitiation complex was pooled and concentrated to 50 µL using Vivaspin 500 centrifugal concentrators. Afterwards, it was incubated with a stoichiometric amount of mtLSU, 5-time excess of mtIF2, Atto390-labeled mRNA, and 5 mM GTP, in initiation buffer for 15 min at 37 °C. For the mtPIC-1 (Fig. 3a), which did not contain fMet-tRNA^Met^_i_, the reaction was supplemented with a five-time excess of Cy3-labeled fMet-tRNA^Met^_i_. The mixtures were analyzed by sucrose gradient centrifugation. The fractions corresponding to the monosome were pooled and concentrated to 50 µL.

### Detection and quantification of Atto390-, Cy3-, and Cy5-labeled species

Fluorescence spectra of Cy3, Cy5, and Atto390 fluorophores were recorded at 25 °C, on a Hitachi Fluorescence Spectrophotometer F-7000, using a 3 mm path length quartz cuvette (105.251-QS, Hellma, Mühlheim, Germany). All spectra were corrected by subtracting the spectra of a reaction in which the nonfluorescent version of the factors (mtIF3, fMet-tRNA^Met^_i_ and mRNA) has been added. The measurements were made using the following settings: Atto390 excitation wavelength 400 nm, emission wavelength 410–650 nm; Cy3 excitation wavelength: 525 nm, emission wavelength: 535–600 nm; Cy5 excitation wavelength: 620 nm, emission wavelength: 625–800 nm.

Calibration curves were made for each of the labeled species, using known concentrations of mtIF3, fMet-tRNA^Met^_i_, and mRNA. Using the calibration curves, we determined the concentrations of Atto390-mRNA, Cy3-fMet-tRNA^Met^_i_, and Cy5-mtIF3 in the initiation reactions from the fluorescence intensity of the samples measured in the same conditions. The calculated concentration of the labeled species was then normalized to the measured concentration of the mtSSU.

For the complete initiation complex, the fraction of monosomes containing bound Cy3-fMet-tRNA^Met^_i_ and Atto390-mRNA was not determined due to the low (≤1 nM) monosome concentration and therefore variable results.

### Confocal fluorescence detection

Fluorescence cross-correlation spectroscopy (FCCS) and single-molecule two-color coincidence detection (TCCD) measurements were performed on a MicroTime 200 confocal microscope (PicoQuant, Berlin, Germany) equipped with two pulsed diode lasers of 510 nm (LDH-D-C-510) and 640 nm (LDH-D-C-640) with an average emission power of 25 µW. All measurements were made on PEG-coated glass slides, to eliminate nonspecific interaction of the sample with a glass slide, using labeled molecules in the concentration range of nanomolar for FCCS and picomolar for TCCD.

The initiation reaction was made as described above, using Cy3-fMet-tRNA^Met^_i_ and Cy5-mtIF3, in the presence of Atto390-labeled or non-labeled mRNA, and the purified initiation complex was analysed with FCCS. In brief, co-diffusion of mtSSU containing both Cy3 and Cy5 fluorophores would result in correlated signals from the Cy3 and Cy5 channels. On the other hand, signals from separately moving labeled particles result in correlation close to zero. As a positive control, i.e., sample with high double-labeled fraction, a 38-nucleotide single stranded DNA sample, labeled at the 3′ end with Cy5 and at the 5′ end with Cy3 was purchased from Sigma–Aldrich. Using TCCD (as previously described in^28^), we determined that in the positive control, 90% of the molecules carry both fluorophores. In addition, a negative control, i.e., sample with no double-labeled molecule containing a mixture of Cy5-labeled mtIF3 and Cy3-labeled fMet-tRNA^Met^_i_ was used.

### In vitro labeling of mitoribosomes at the accessible lysines

Mitoribosomes (200 nM) were incubated with a 10-time excess (2 µM) of NHS functionalized dyes (Atto488 or Cy5) in Labeling Buffer (50 mM HEPES-KOH pH 7.5, 30 mM KCl, 20 mM MgCl_2_) at 37 °C for 20 min. To purify the labeled ribosomes from the excess of free dye, the labeling reaction was overlaid on top of 1 mL sucrose cushion in Labeling Buffer and centrifuged at 55,000 rpm for 17 h at 4 °C in a TLA-100.4 rotor. The pellet consisting of labeled ribosomes was used fresh for further experiments.

### Double labeling of ribosomes for optical tweezers experiments

Samples containing pellets of Atto488- and Cy5-labeled mitoribosomes prepared as described above were dissolved in Dissociation Buffer for 3 h at 4 °C, overlaid on top of a 10–30% sucrose gradient prepared in Dissociation Buffer and centrifuged at 21,000 rpm for 17 h at 4 °C in an SW41 Ti rotor. The gradients were then fractionated using a Biocomp Fractionator, and the fractions containing the labeled subunits were collected separately and centrifuged at 55,000 rpm for 17 h in an SW60 Ti rotor. The pellet corresponding to Cy5-mtSSU was dissolved in Initiation Buffer and used fresh for optical tweezers experiments. The pellets corresponding to the Atto488 mtSSU and Cy5 mtLSU were dissolved in Reassociation Buffer, mixed in an equimolar ratio and incubated at 4 °C for 1 h to allow the reassociation of the subunits. The sample was then overlaid of top of a 10–30% sucrose gradient and centrifuged at 21,000 rpm for 17 h at 4 °C in an SW41 Ti rotor to separate the reassociated mitoribosomes from the non-reassociated subunits. After fractionation of the gradient, the double-labeled monosomes were pelleted by centrifugation at 55,000 rpm in a TLA-100.4 rotor for 17 h at 4 °C. The resulting pellet was dissolved in Initiation Buffer and used fresh for optical tweezers experiments.

### Preparation of DNA/mRNA constructs for optical tweezers experiments

The two mRNAs used for optical tweezers experiments were purchased from Eurofins as DNA/RNA chimeras, containing at the 3′ end the cohesive BmtI sequence GATC, followed by the first 58 nucleotides of the native human Cox2 mRNA sequence, and the same sequence without the start codon. Biotinylated lambda DNA was prepared using Klenow Polymerase, biotin-dCTP and biotin dATP as previously described^29,30^ and digested with BmtI for 2 h at 37 °C. After gel electrophoresis using a 1% agarose gel, the 35 kbp construct was purified and ligated with the DNA/RNA chimeras for 1 h at room temperature. The ligated product was separated with gel electrophoresis, purified and stored at 4 °C no longer than two days, in the presence of RNase inhibitors.

### Single-molecule imaging in an optical tweezer setup

Optical tweezer experiments were performed on C-TrapTM system integrating optical tweezers, confocal fluorescence microscopy, and microfluidics and recorded using BlueLake software (LUMICKS B.V., the Netherlands). Initiation reactions were made by incubating 200 nM double-labeled ribosomes or Cy5-labeled mtSSU, 240 nM mtIF3, 240 nM mtIF2, 240 nM fMet-tRNA^Met^_i_, and 400 nM biotinylated DNA/RNA construct in Initiation Buffer for 2 h at 37 °C. Tether formation was performed in situ (inside the laminar flow cell chamber, LUMICKS) by trapping 4.47 µm streptavidin-coated polystyrene beads with optical traps in Channel 1 and bringing beads in contact with DNA/RNA constructs, preincubated with proteins, in Channel 2. Binding of the complete mitoribosome or the small subunit to RNA was detected by dual-color imaging with 532 nm and 638 nm lasers in Initiation Buffer (Channel 3). Images were further converted to *.tiff and analyzed with an open source Java image processing program ImageJ (https://imagej.net/ImageJ).

### Reporting summary

Further information on research design is available in the Nature Research Reporting Summary linked to this article.

## Supplementary information

Supplementary Information

Peer Review File

Reporting Summary

## Data Availability

The data that support this study is available from the corresponding authors upon reasonable request. The cryo-EM maps have been deposited in the Electron Microscopy Data Bank with accession codes EMD-10021, EMD-10022, EMD-10023, EMD-10024, EMD-10025, EMD-10026, EMD-10027, EMD-10028, EMD-10029, EMD-10030, EMD-10031 and EMD-10032. The atomic models have been deposited in the Protein Data Bank under accession codes PDB 6RW4 and PDB 6RW5. The data source behind Fig. 6d, Supplementary Fig. 9a, b and Supplementary Table 4 are provided as a Source Data file.

## Source Data

Source Data
