# Supplementary material for: Distinct pre-initiation steps in human mitochondrial translation
Source: Nat Commun. 2020 Jun 10;11:2932. doi: 10.1038/s41467-020-16503-2 (PMC7287080; doi:10.1038/s41467-020-16503-2)
Supplement: Supplementary file 1 — Supplementary Information [file 41467_2020_16503_MOESM1_ESM.pdf]

SUPPLEMENTARY INFORMATION

Distinct pre-initiation steps in human mitochondrial translation

Khawaja, Itoh et al.

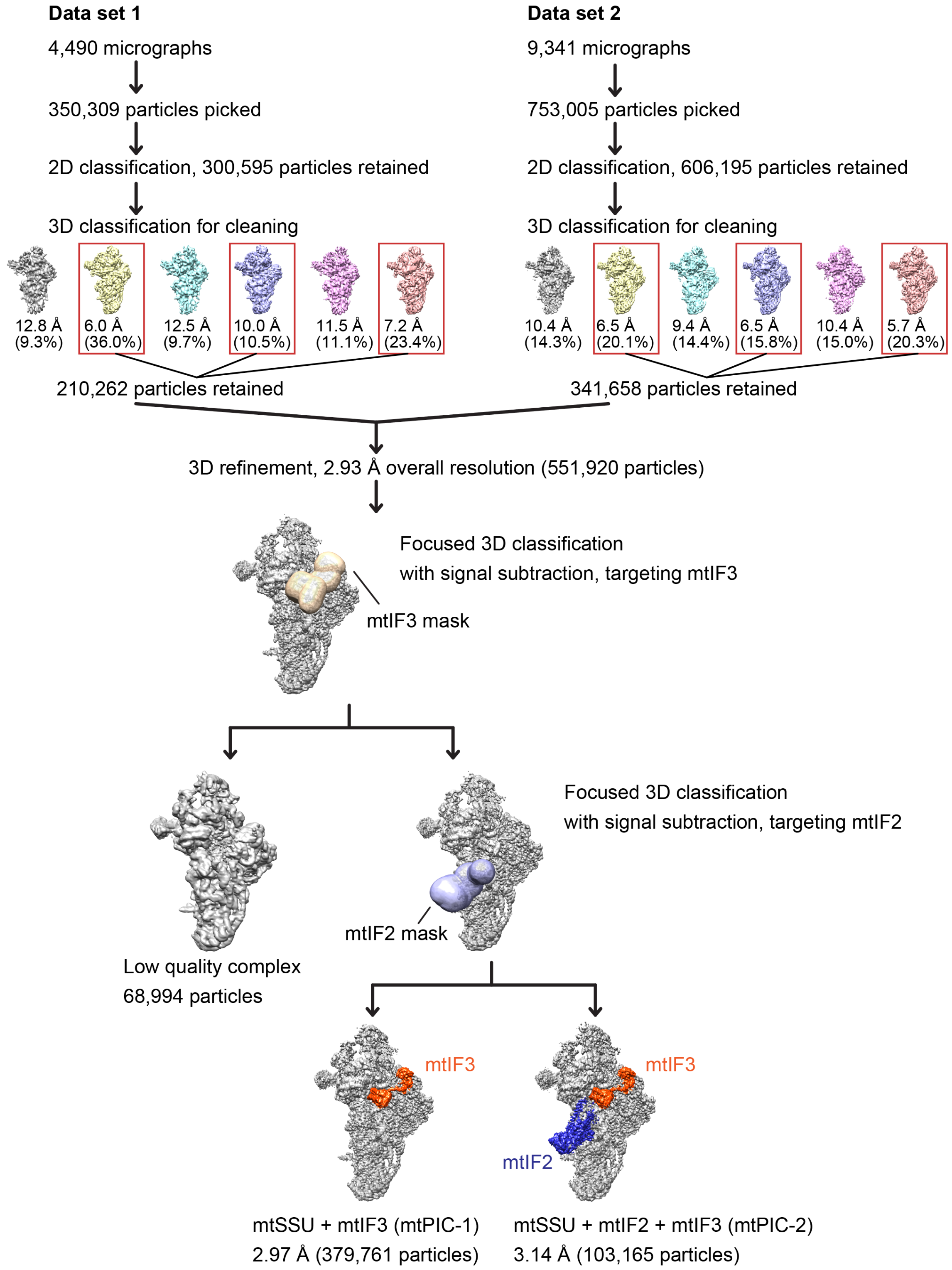

Supplementary Figure 1. Data processing overview.
Two data sets were processed separately up to 3D classification for cleaning. After merging the data and 3D refinement, two steps of focused 3D classification with signal subtraction were performed by using mtIF3 and mtIF2 masks to separate the mtIF3-only and mtIF2-mtIF3 bound complexes.

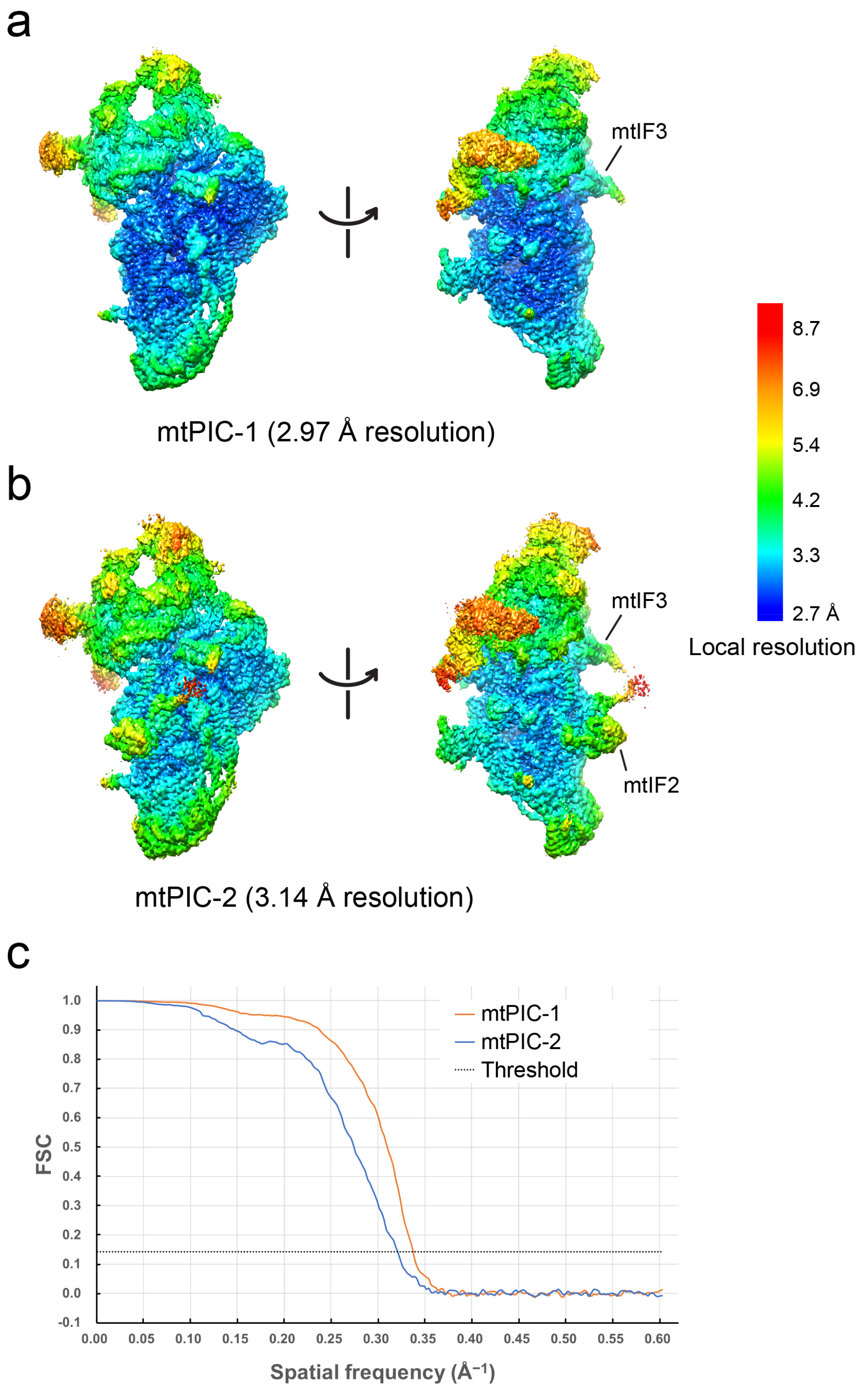

Supplementary Figure 2. Resolution of the maps.
a,b Local resolution was estimated by Relion 3 and presented by color gradation on the surfaces of the unsharpened overall maps of mtPIC-1 **(a)** and mtPIC-2 **(b)**. **c** Gold-standard Fourier shell correlation (FSC) curves of the maps were colored orange (mtPIC-1) and blue (mtPIC-2), respectively with the threshold 0.143.

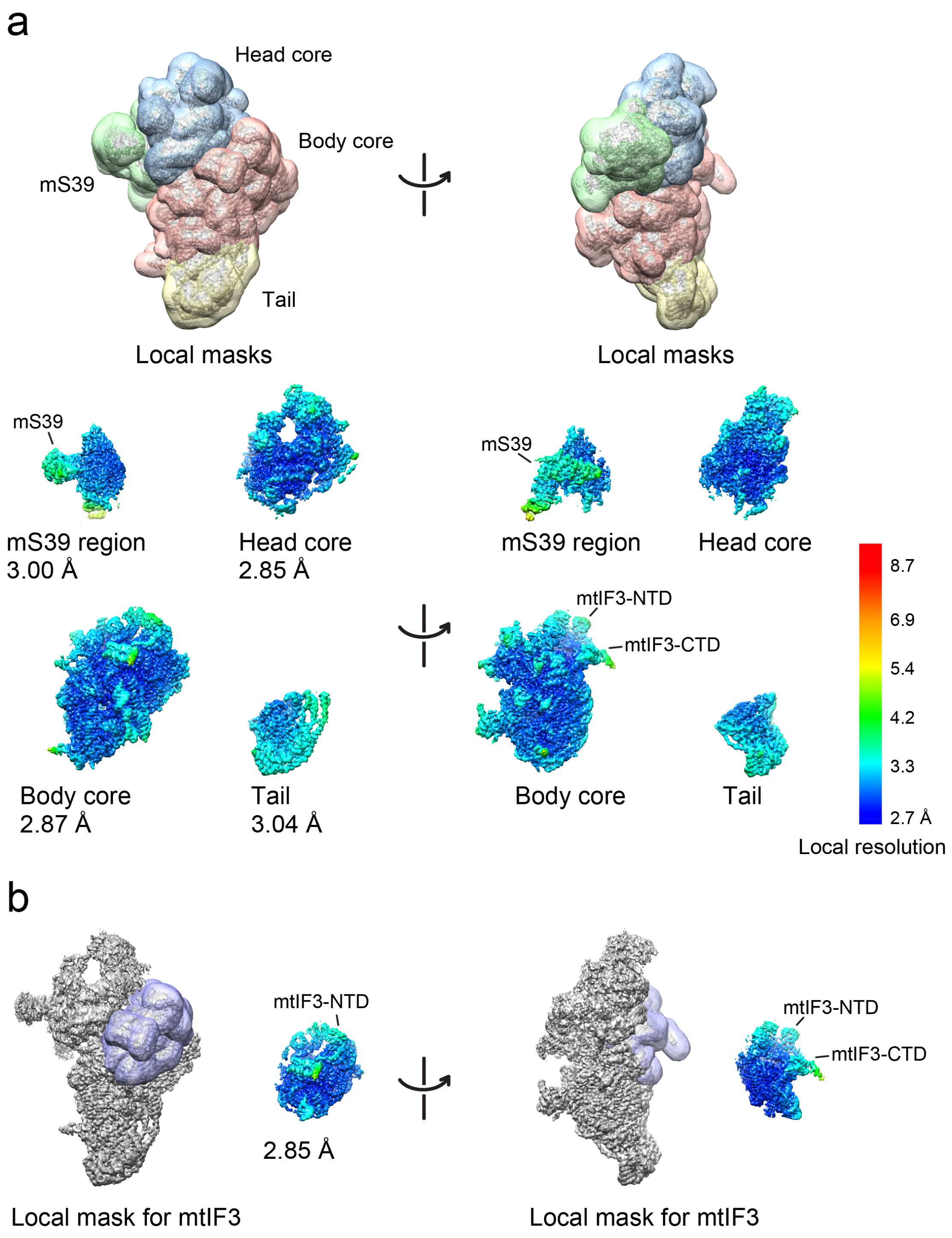

Supplementary Figure 3. Local masked refinement of mtPIC-1.
a Four local masks covering the body core, the head core, tail, and the mS39 region, respectively, were prepared and used for local 3D refinement to improve the local resolution. Local resolution was estimated by Relion 3 and presented by color gradation on the surfaces of the unsharpened maps. **b** A local mask covering mtIF3 and its neighboring was prepared to improve the local resolution of mtIF3. The resulting local resolution is shown.

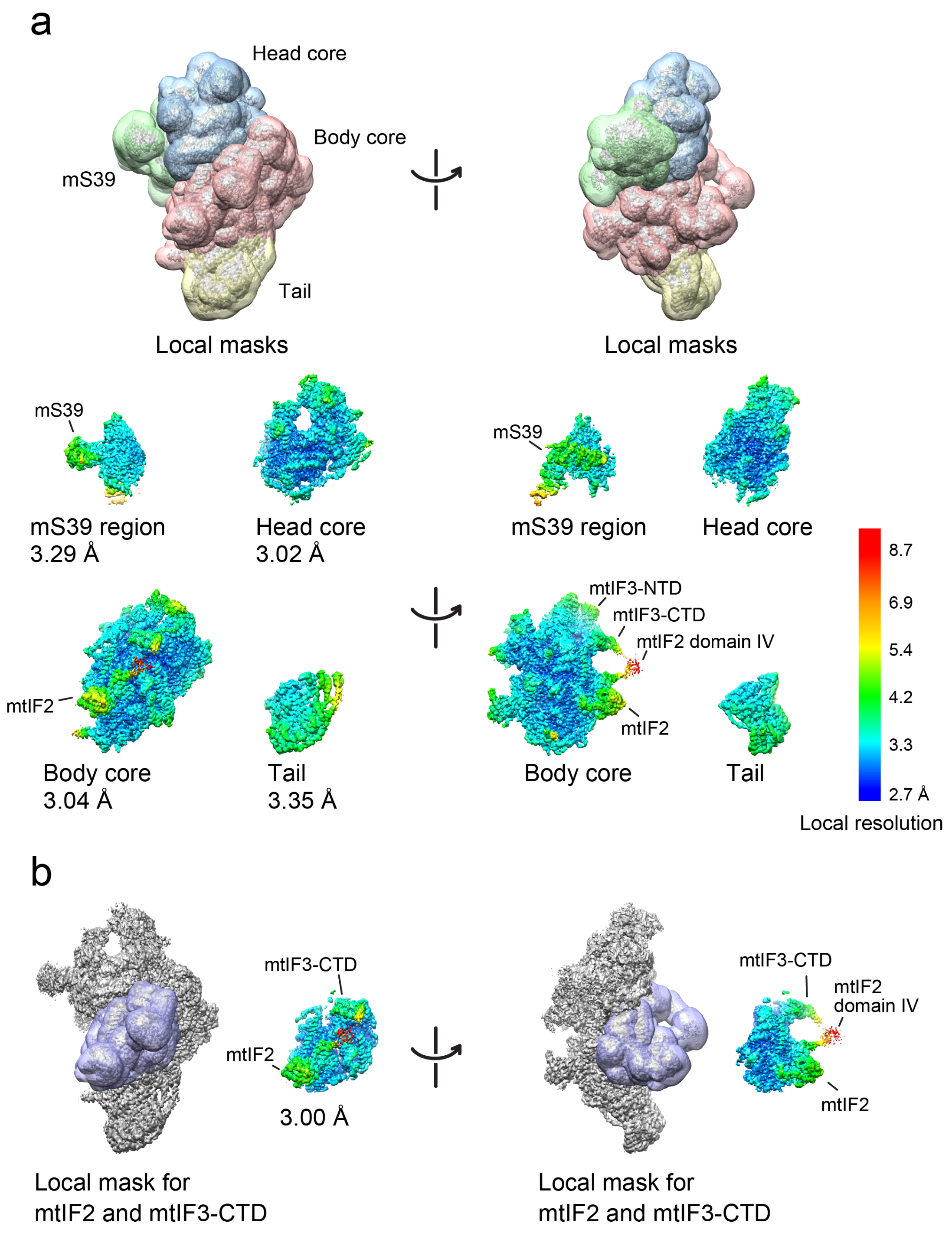

Supplementary Figure 4. Local masked refinement of mtPIC-2.
a Four local masks covering the body core, the head core, tail, and the mS39 region, respectively, were prepared and used for local 3D refinement to improve the local resolution. Local resolution was estimated by Relion 3 and presented by color gradation on the surfaces of the unsharpened maps. **b** A local mask covering mtIF2, mtIF3-CTD, and its neighboring was prepared to improve the local resolution of mtIF2 and mtIF3-CTD. The resulting local resolution is shown.

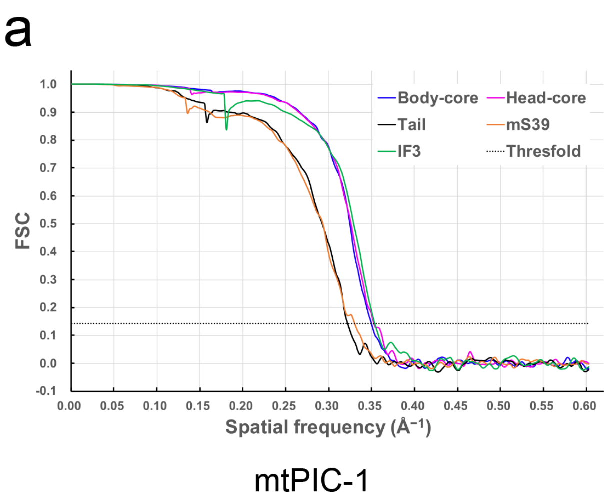

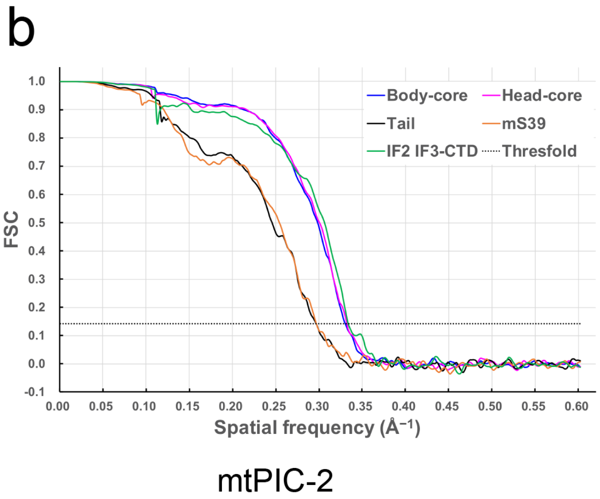

Supplementary Figure 5. FSC curves of the local masked refined maps.
Gold standard FSC curves of the local masked refined maps for mtPIC-1 **(a)** and mtPIC-2 **(b)** are shown with the threshold 0.143.

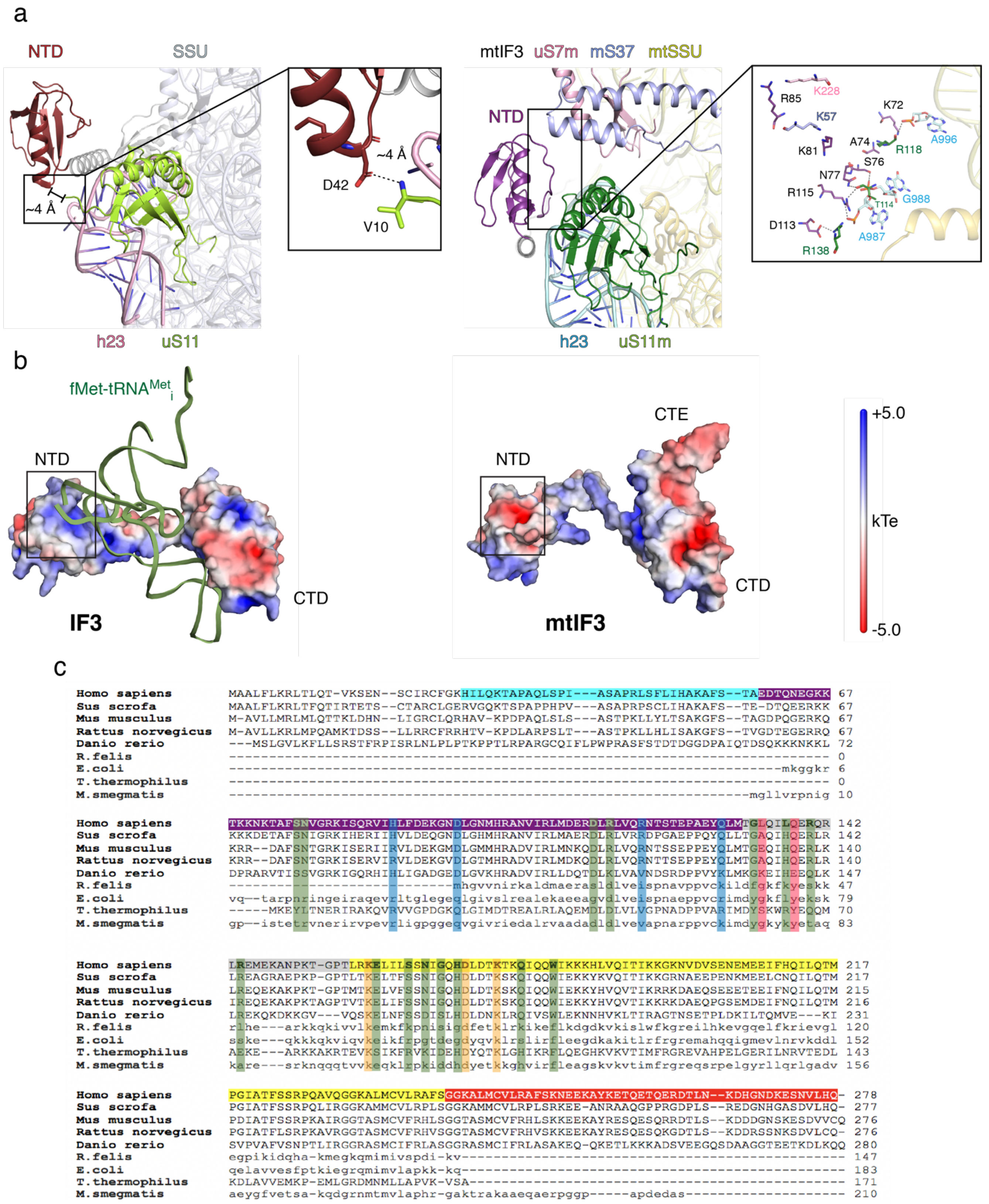
a
NTD
SSU
~4 Å
~4 Å
D42
V10
h23
uS11
mtIF3
uS7m
mS37
mtSSU
NTD
R85
K228
K57
K72
K81
A74
R118
A996
S76
N77
R115
G988
T114
D113
A987
R138
h23
uS11m
b
fMet-tRNA^Met_i
NTD
IF3
CTD
NTD
CTE
CTD
mtIF3
+5.0
kTe
-5.0
c
Homo sapiens MAALFLKRLTLQT-VKSEN--SCIRCFGKHILQKTAPAQLSPI---ASAPRLSFLIHAKAFS--TAEDTQNEGKK 67
Sus scrofa MAALFLKRLTFQTIRTETS--CTARCLGERVGQKTSPAPPHPV---ASAPRPSCLIHAKAFS--TE-DTQEERKK 67
Mus musculus M-AVLLMRLMLQTTKLDHN--LIGRCLQRHAV-KPDPAQLSLS---ASTPKLLYLTSAKGFS--TAGDPQGERKQ 67
Rattus norvegicus M-AVLLKRLMPQAMKTDSS--LLRRCFRRHTV-KPDLARPSLT---ASTPKLLHLISAKGFS--TVGDTEGERRQ 67
Danio rerio ---MSLGVLKFLLSRSTFRPISRLNLPLPTKPPTLRPARGCQIFLPWPRASFSTDTDGGDPAIQTDSQKKKNKKL 72
R.felis --- 0
E.coli ---mkggkr 6
T.thermophilus --- 0
M.smegmatis ---mgllvrpnig 10
Homo sapiens TKKNKTAFSNVGRKISQRVIHLFDEKGNDLGNMHRANVIRLMDERDLRLVQRNTSTEPAEYQLMTGLQILQERQR 142
Sus scrofa KKKDETAFSNIGRKIHERIIHVLDEQGNDLGHMHRANVIRLMAERDLRLVRRDPGAEPPQYQLLTGAQIHQERLR 142
Mus musculus KRR--DAFSNTGRKISERIIRVLDEKGMDLGMMHRADVIRLMNKQDLRLVQRNTSSEPPEYQLMTGEQIHQERLK 140
Rattus norvegicus KRR--DAFSNTGRKISERVIRVLDEKGVDLGTMHRADVIRLMDKQDLRLVQRNTTSEPPEYQLMTGAQIHQERLR 140
Danio rerio DPRARVTISSVGRKIGQRHIHLIGADGEDLGVKHRADVIRLLDQTDLKLVAVNDSRDPPVYKLMKGKEIHEEQLK 147
R.felis ----------------------------mhgvvnirkaldmaerasldlveispnavppvckildfgkfkyeskk 47
E.coli vq--tarpnringeiraqevrltglegeqlgivslrealekaeeagvdlveispnaeppvcrimdygkflyeksk 79
T.thermophilus -----MKEYLTNERIRAKQVRVVGPDGKQLGIMDTREALRLAQEMDLDLVLVGPNADPPVARIMDYSKWRYEQQM 70
M.smegmatis gp--istetrvnerirvpevrligpggeqvgivriedalrvaadadldlvevapnarppvckimdygkykyetaq 83
Homo sapiens LREMEKANPKT-GPTLRKELILSSNIGQHDLDTKTKQIQQWIKKKHLVQITIKKGKNVDVSENEMEEIFHQILQTM 217
Sus scrofa LREAGRAEPKP-GPTLTKELTFSSNIGQHDLDTKSKQIQQWIEKKYKVQITIKKGRNAEEPENKMEELCNQILQTM 217
Mus musculus LREQEKAKPKT-GPTMTKELVFSSNIGQHDLDTKSKQIQQWIEKKYHVQVTIKRRKDAEQSEEETEEIFNQILQTM 215
Rattus norvegicus IREQEKAKPKTAGPTVTKELIFSSNIGQHDLDTKSKQIQQWIEKKYHVQVTIKKRKDAEQPGSEMDEIFNQILQTM 216
Danio rerio LREKQKDKKGV---VQSKELNFSSDISLHDLDNKLRQIVSWLEKNNHVKLTIRAGTNSETPLDKILTQMVE---KI 231
R.felis rlhe---arkkqkivvlkemkfkpnisigdfetklrkikeflkdgdkvkislwfkgreilhkevgqelfkrievgl 120
E.coli sske---qkkkqkviqvkeikfrpgtdegdyqvklrslirfleegdkakitlrfrgremahqqigmevlnrvkddl 152
T.thermophilus AEKE---ARKKAKRTEVKSIKFRVKIDEHDYQTKLGHIKRFLQEGHKVKVTIMFRGREVAHPELGERILNRVTEDL 143
M.smegmatis kare---srknqqqtvvkeqklrpkiddhdyetkkghvirfleagskvkvtimfrgreqsrpelgyrllqrlgadv 156
Homo sapiens PGIATFSSRPQAVQGGKALMCVLRAFSGGKALMCVLRAFSKNEEKAYKETQETQERDTLN--KDHGNDKESNVLHQ- 278
Sus scrofa PGIATFSSRPQLIRGGKAMMCVLRPLSGGKAMMCVLRPLSRKEE-ANRAAQGPPRGDPLS--REDGNHGASDVLHQ- 277
Mus musculus PDIATFSSRPKAIRGGTASMCVFRHLSGGTASMCVFRHLSKKEEKAYRESQESQRRDTLS--KDDDGNSKESDVVCQ 276
Rattus norvegicus PGIATFLSRPKAVRGGTASMCVFRHVSGGTASMCVFRHVSKKEEKAYRESQESQKGDTLS--KDDRSSNKSDVLCQ- 276
Danio rerio SVPVAFVSNPTLIRGGRASMCIFRLASGGRASMCIFRLASAKEQ-QKETLKKKADSVEEGQSDAAGGTEETKDLKQQ 280
R.felis egpikidqha-kmegkqmimivspdi-kv-- 147
E.coli qelavvesfptkiegrqmimvlapkk-kq-- 183
T.thermophilus KDLAVVEMKP-EMLGRDMNMLLAPVK-VSA--- 171
M.smegmatis aeygfvetsa-kqdgrnmtmvlaphr-gaktrakaaeqaerpggp----apdedas--------------------- 210

Supplementary Figure 6. Comparison with bacterial IF3.
a Comparison of the IF3 NTD and the small ribosomal subunit interactions. IF3-NTD of the bacterium *Thermus thermophilus* makes a possible interaction with the uS11 (green) of SSU (PDB; 5LMN). The closest distance between residues of the NTD (D41) and uS11 (V10) is marked. In contrast, mtIF3 is associated to the mtSSU platform through several interactions with h23 (cyan) of 12S rRNA and uS11m (dark green, in close proximity to uS7m (pink) and mS37 (blue). **b** Comparison of the electrostatic potential on the surfaces of the bacterial IF3 (PDB: 5LMS, shown with fMet-tRNA$^{Met}_{i}$) and mtIF3. Electrostatic potential was calculated by using ABPS [29]. Electronegative and electropositive regions are colored in red and blue, respectively. The binding site of the IF3-NTD with the elbow of fMet-tRNA$^{Met}_{i}$ is marked by a rectangle. **c** Sequence alignment of the human mtIF3 with its vertebrate and bacterial orthologs. The human mtIF3 sequence is color-coded corresponding to its domain organization (NTE; turquoise, NTD; purple, CTD; yellow, CTE; red). Green and orange columns indicate the binding sites of mtIF3 with the mtSSU. The green columns represent mtIF3 residues which are not conserved while the orange columns display the conserved residues. The pink columns show residues involved in the tRNA discrimination in bacteria (G71 and Y75), which are not conserved in vertebrates. Residues that bind the elbow of initiator tRNA in bacteria are displayed in blue.

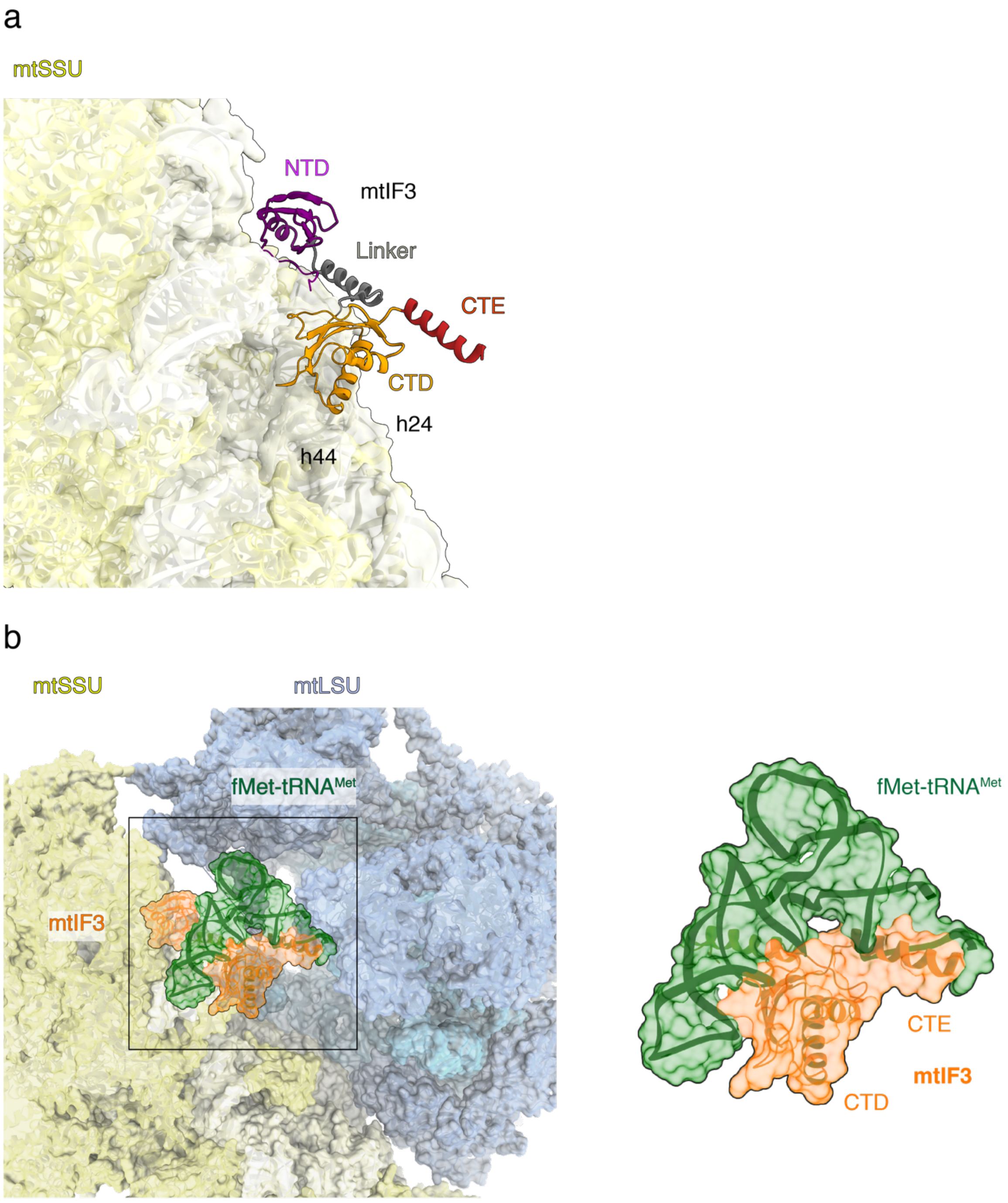

Supplementary Figure 7. The mtIF3-CTE prevents binding of the fMet-tRNA$^{Met}{}_{i}$ to the mtSSU.

a The mitochondrial specific mtIF3-CTE forms a helix facing outward from the mtSSU. **b** The mtIF3-CTE occupies the region of the fMet-tRNA$^{Met}{}_{i}$ acceptor stem binding site shown by superposition with the complete initiation complex (PDB: 6GAW). The transparent surface and ribbon representation of the factors (right panel) illustrate the clash of mtIF3-CTD and CTE with fMet-tRNA$^{Met}{}_{i}$ indicative of their alternate association to the mtSSU.

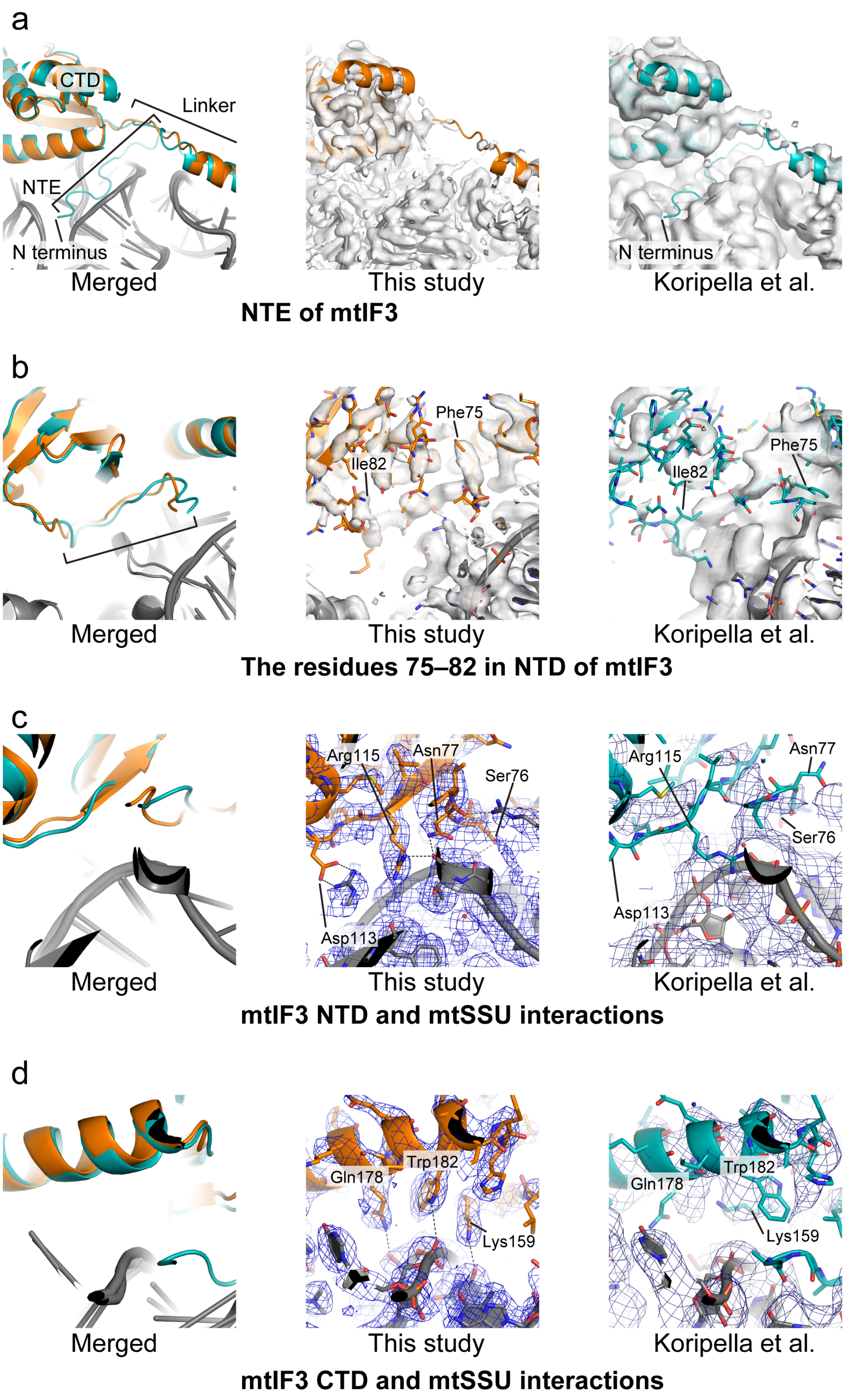
a
CTD
Linker
NTE
N terminus
Merged
This study
N terminus
Koripella et al.
NTE of mtIF3
b
Phe75
Ile82
Phe75
Ile82
Merged
This study
Koripella et al.
The residues 75–82 in NTD of mtIF3
c
Arg115
Asn77
Ser76
Asp113
Arg115
Asn77
Ser76
Asp113
Merged
This study
Koripella et al.
mtIF3 NTD and mtSSU interactions
d
Gln178
Trp182
Lys159
Trp182
Gln178
Lys159
Merged
This study
Koripella et al.
mtIF3 CTD and mtSSU interactions

Supplementary Figure 8. Comparison of the mtIF3-mtSSU interactions between this study and Koripella et al. [6].

a The NTE of mtIF3 modeled in Koripella et al.[6]. The merged structures of mtIF3 from this report (orange) and from Koripella et al. (green cyan) are shown in the left panel. The structures are superposed by using the densities of the mtSSU body for fitting. The cryo-EM densities of this report (middle panel) and Koripella et al. (right panel, EMD-9358) are shown as surface with the contour levels of 6.0 and 3.6, respectively. The NTE is modeled only in Koripella et al., whereas it is missing in our model. There is no significant density of the NTE in either of the maps. **b** The loop consists of the residues 75–82 in NTD. Superposed ribbon models (left panel) and additional stick models with the cryo-EM densities of this report (middle panel) and Koripella et al. (right panel) are shown with the contour levels of 5.0 and 3.0, respectively. In our model, all side chains in this region are supported by the density, while some of the side chains do not agree with the density in Koripella et al. This causes the different interpretation of the interactions between the mtIF3-NTD and the mtSSU. **c** Picked-up interactions between the mtIF3-NTD and the mtSSU. Merged ribbon model (left panel) and the individual stick models with polar interactions (middle and right panels) are shown. The cryo-EM densities are shown as mesh with the contour levels of 5.0 and 3.0, respectively in the middle and right panels. Ser76, Asn77, Arg111, and Asp113 are interacting in our model, whereas they are away from the mtSSU in Koripella et al. **d** Picked-up interactions between the mtIF3 CTD and the mtSSU. Merged ribbon model (left panel) and the individual stick models and polar interactions (middle and right panels) are shown. The cryo-EM densities are shown as mesh with the contour levels of 9.0 and 6.0, respectively in the middle and right panel. Interactions through Lys159, Gln178, and Trp182 are supported by the density in our model. In Koripella et al., the Gln178 side chain is out of the density, the Lys159 side chain occupies the density for Trp182 and the Trp182 side chain is kicked out.

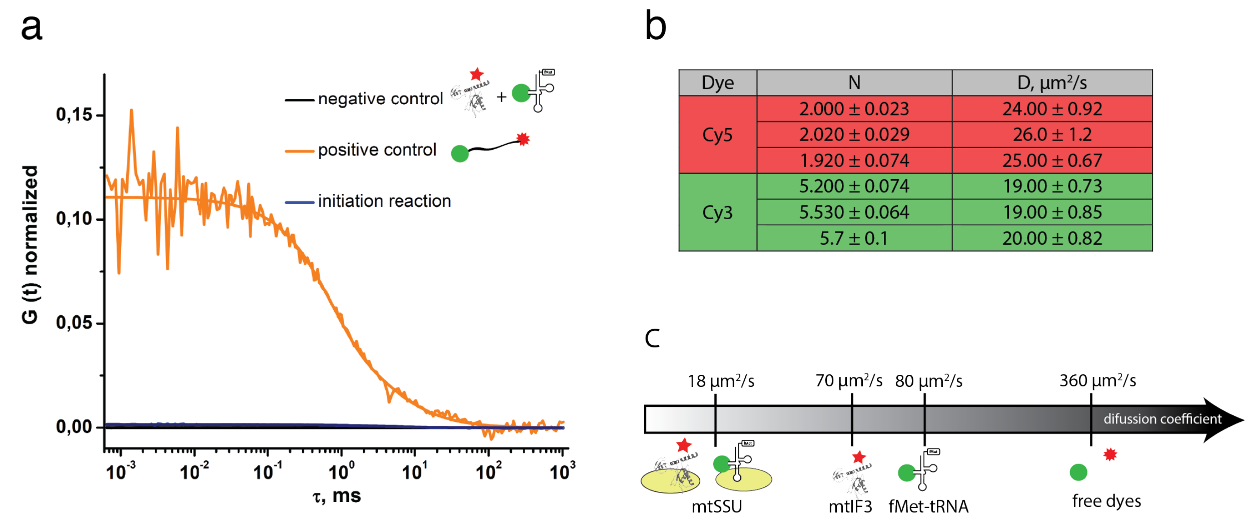

Dye	N	D, μm²/s
Cy5	2.000 ± 0.023	24.00 ± 0.92
	2.020 ± 0.029	26.0 ± 1.2
	1.920 ± 0.074	25.00 ± 0.67
Cy3	5.200 ± 0.074	19.00 ± 0.73
	5.530 ± 0.064	19.00 ± 0.85
	5.7 ± 0.1	20.00 ± 0.82

Supplementary Figure 9. Fluorescence cross-correlation spectroscopy measurements of the mtPIC formed with Cy3-fMet-tRNA$^{Met}_{i}$ and Cy5-mtIF3.

a Cross-correlation curves normalized to concentration, for the mtPIC (blue curve), negative control – a mixture of Cy5-mtIF3 and Cy3-fMet-tRNA$^{Met}_{i}$ (black curve) and positive control – a 38-nucleotide DNA labeled with Cy3 at the 3′ end and with Cy5 at the 5′ end (orange curve). **b** Diffusion coefficients of the Cy3 and Cy5 labeled species during the measurement time of 15 minutes show a constant value of 20 μm^2/s, proving that the labeled mtIF3 and fMet-tRNA$^{Met}_{i}$ do not dissociate from the complex formed with the mtSSU. Diffusion coefficients for Cy3 and Cy5 channels show slightly different values due to mismatch of the green and red detection volumes in confocal microscope. **c** Expected diffusion coefficients of the participating labeled species. Source data are provided as a Source Data file.

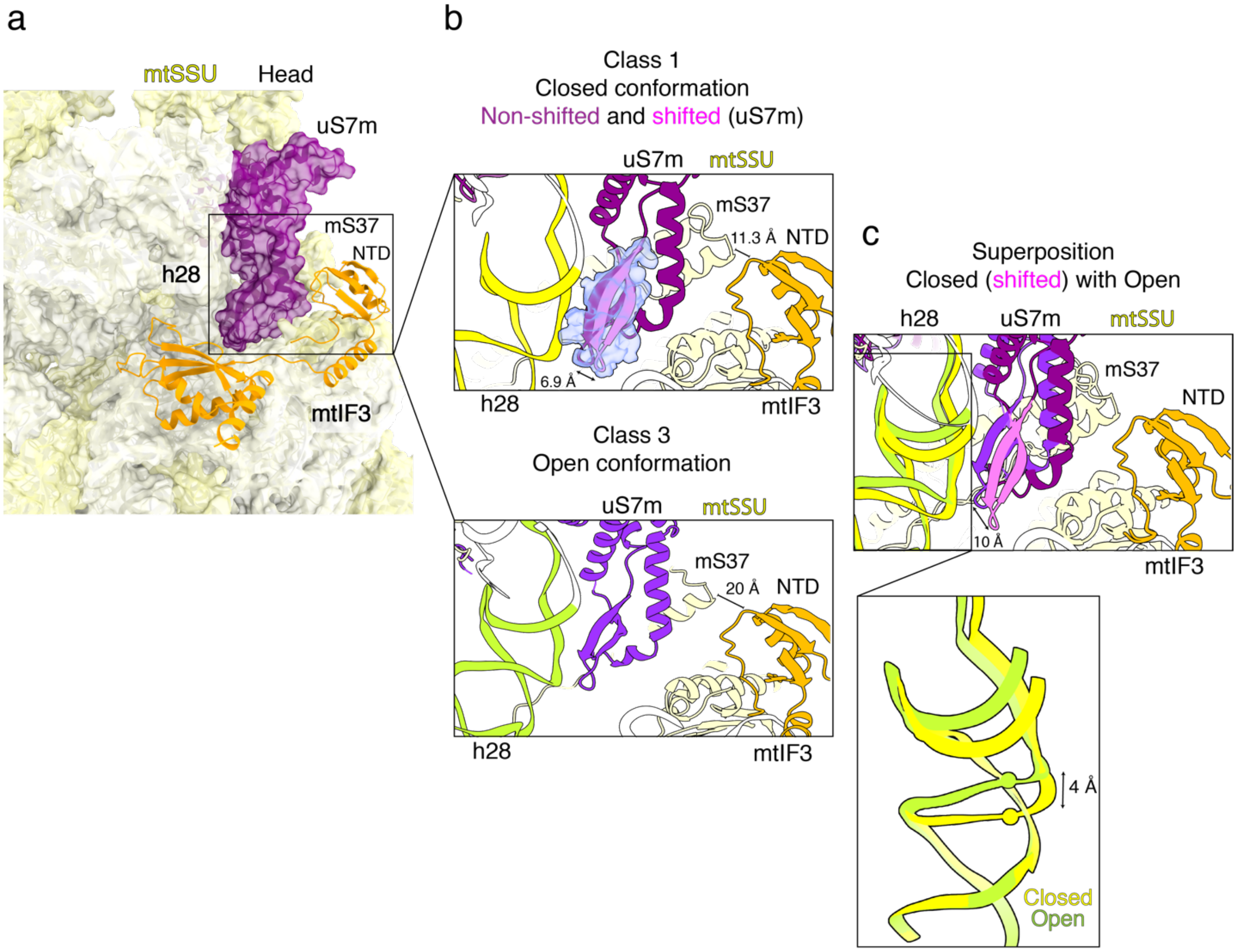

Supplementary Figure 10. Conformations of the h28-associated region of uS7m during the mtSSU head movement of the mtPIC-1.

a Overview of the uS7m (violet) bound to the mtSSU head (yellow) located adjacent to the mtIF3 (orange). **b** Zoom-in panels illustrate the mtSSU head orientation for class 1 (closed) and class 3 (open) of the mtPIC-1. In class 1, two alternative conformations, non-shifted and shifted, are observed for the β hairpin of uS7m, which are colored purple and pink, respectively. The density map of the β hairpin is shown. In the closed conformation, mS37 is located in close vicinity of the mtIF3-NTD, whereas it moves away from the mtIF3-NTD in the open head conformation (class 3). **c** Superposition of the closed (shifted) and open state of the mtSSU head exhibits a movement of 10 Å of G165 in the β hairpin of uS7m. The h28 of 12S rRNA in closed (yellow) and open (green) states is highlighted. Positioning of U1468 of the h28 in both states is shown as spheres.

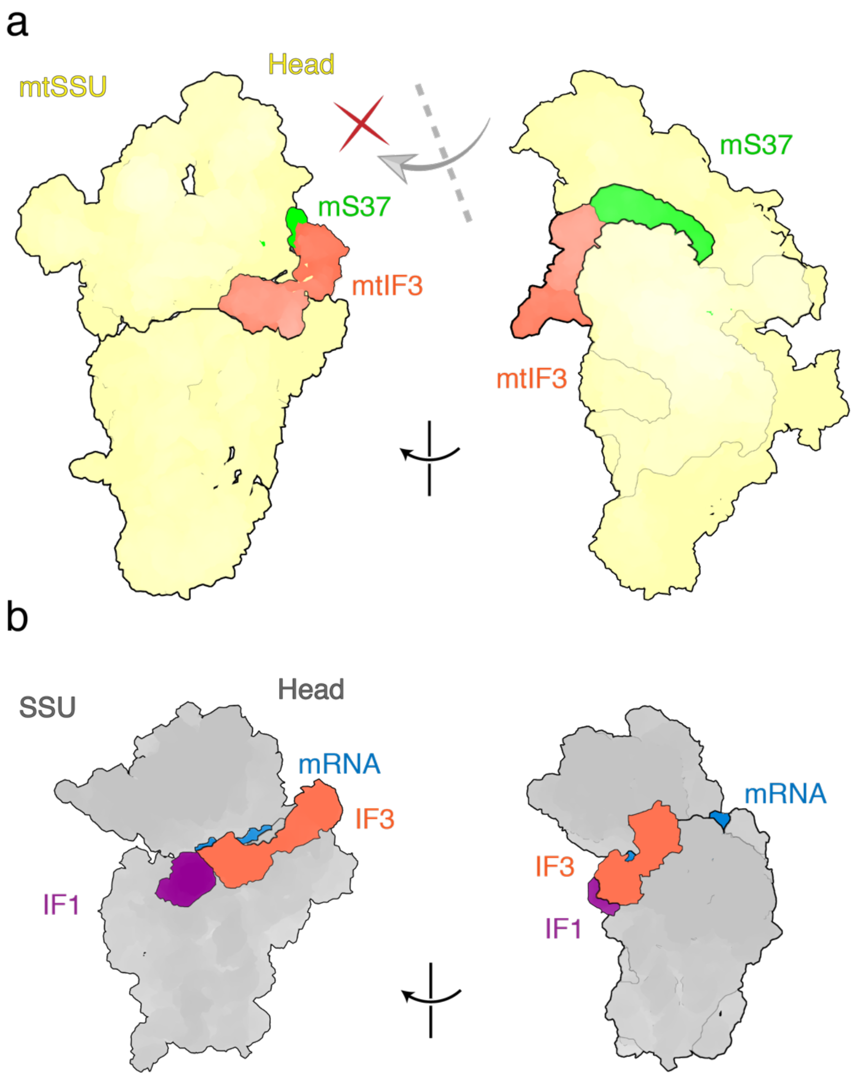

Supplementary Figure 11. mS37 and mtIF3 coordinate movement of the mtSSU head.
a The conformation of the mtSSU head is coordinated by the synergistic action of mS37 and mtIF3. mS37 lies in proximity to the mtIF3-NTD at the mtSSU platform, and cooperatively they restrict the rotation of mtSSU head towards more closed conformation that would clash with mtIF3 (the axis of rotation is shown by dotted line and gray arrow, the limitation of head movement is represented by red cross). **b** In bacterial system, the restricted SSU head movement and accommodation of IF2 is supported by IF3-IF1 interactions.

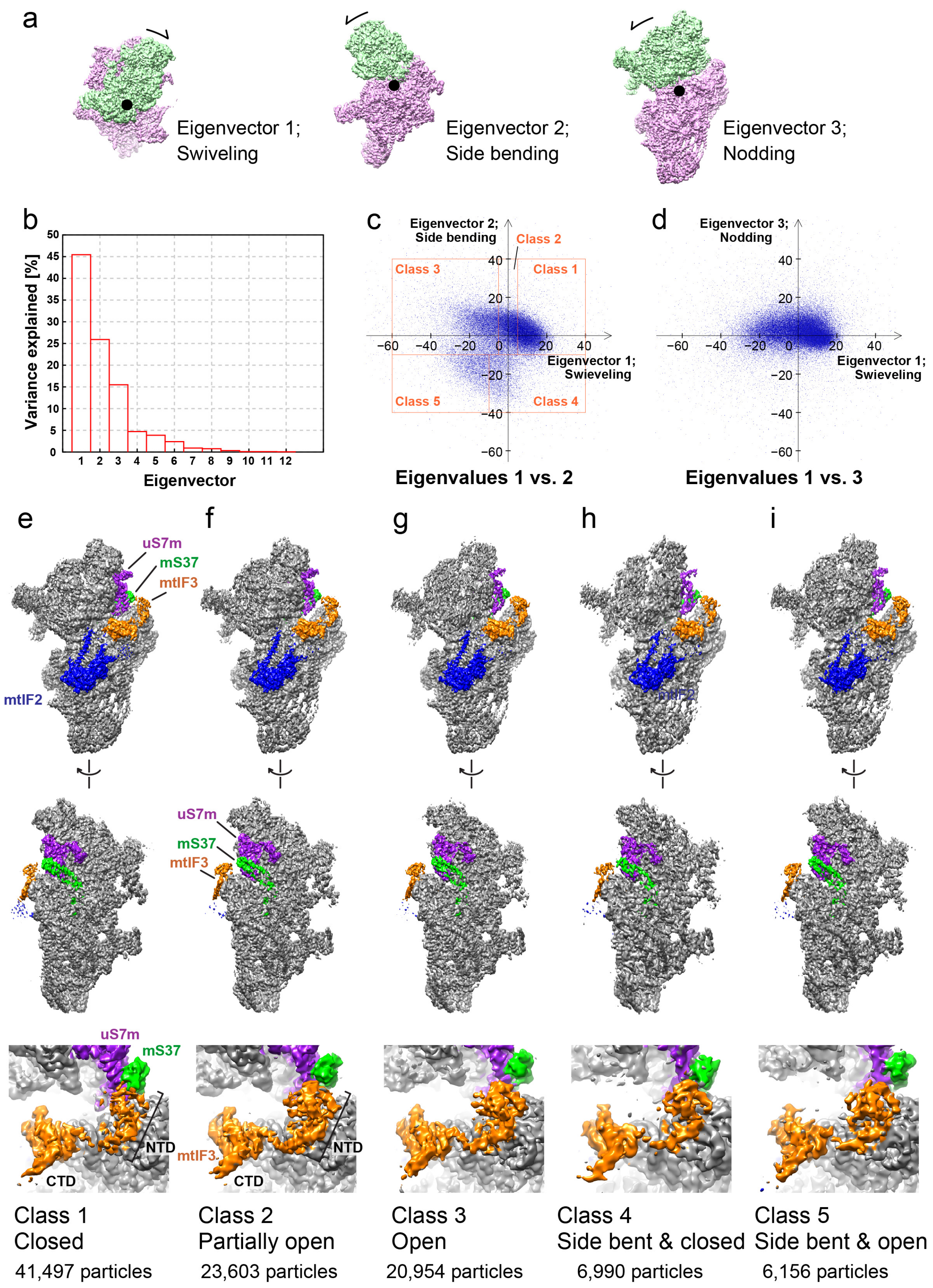
a
Eigenvector 1;
Swiveling
Eigenvector 2;
Side bending
Eigenvector 3;
Nodding
b
Variance explained [%]
Eigenvector
c
Eigenvector 2;
Side bending
Class 2
Class 3
Class 1
Class 5
Class 4
Eigenvector 1;
Swieveling
Eigenvalues 1 vs. 2
d
Eigenvector 3;
Nodding
Eigenvector 1;
Swieveling
Eigenvalues 1 vs. 3
e
f
g
h
i
uS7m
mS37
mtIF3
mtIF2
NTD
CTD
Class 1
Closed
41,497 particles
Class 2
Partially open
23,603 particles
Class 3
Open
20,954 particles
Class 4
Side bent & closed
6,990 particles
Class 5
Side bent & open
6,156 particles

Supplementary Figure 12. Head motion analysis of mtPIC-2.
3D multibody analysis of the mtPIC-2 using the head and body masks (mtIF2 and mtIF3 are included in the body mask), similar in Fig. 3. Principal component analysis was done and the 1st to 3rd eigenvectors are mainly contributed by the head rotation, illustrated in **a**. The head and body are colored light green and pink, respectively. The rotation axis and the orientation are shown as a dot and an arrow, respectively. **b** Histogram of the variances explained by the 12 eigenvectors. **c, d** The eigenvalues (vectors 1, 2 and 3) of randomly chosen 100,000 particles are plotted two-dimensionally, against each other. Each particle is shown as a blue dot. The motions of the eigenvectors 1 (swiveling) and 2 (side bending) have non-gaussian distribution. Unlike in mtPIC-1, there are two peaks for the side bending motion. The particles are separated into five classes, based on the 1st and 2nd eigenvalues. Rectangle zones used for the classification are shown in **d**. **e-i** Classified particles are aligned to reconstitute their density maps. mtIF3, uS7m, and mS37 are colored orange, purple, and green, respectively. Similar to mtPIC-1, in the closed conformation, the density of the mtIF3-NTD is weaker and mS37 (green) and uS7m (purple) from the head contact to the mtIF3-NTD, suggesting the destabilization of the domain.

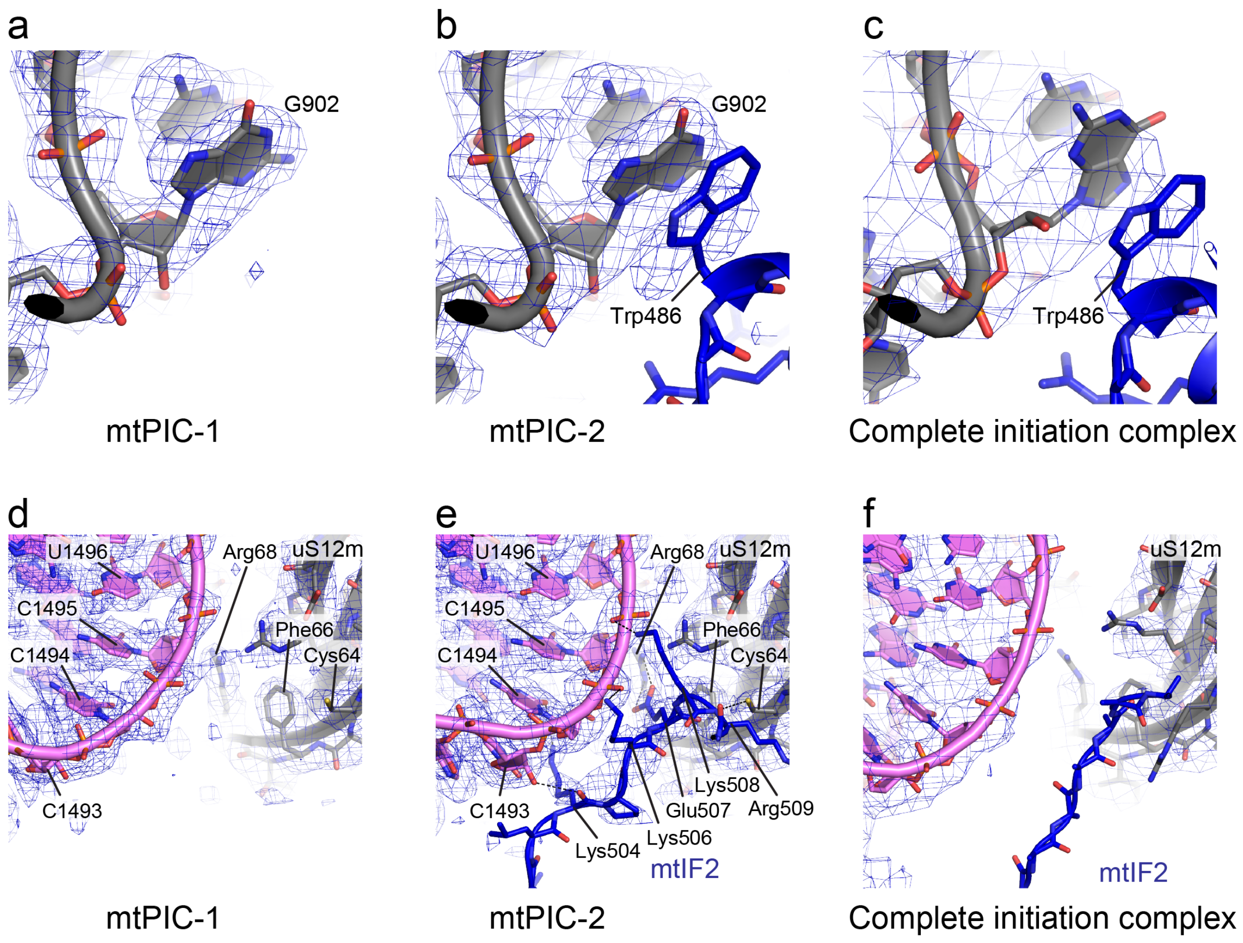

Supplementary Figure 13. Comparison of the mtIF2 insertion domain and its interactions with the mtSSU between the mtPIC-2 and the complete initiation complex.

a-c Zoomed-in views of G904 of 12S rRNA from mtPIC-1 (**a**) and mtPIC-2 (**b**), and the corresponding nucleotide from the complete initiation complex (**c**, PDB 6GAZ [3]). mtIF2 is colored blue. The cryo-EM densities are shown with the contour levels of 6.0, 6.0, and 7.0 (EMD-4369 [3]), respectively. Trp486 from mtIF2 colored blue is stacking on G904, although the density is weak except for the indole ring. The densities support that G904 is in typical sugar and base conformations, i.e. C3′-endo and anti-conformations, respectively, in mtPIC-1 (**a**) and mtPIC-2 (**b**). In contrast, the corresponding nucleotide is modeled as C2′-endo and syn-conformations in the complete initiation complex (**c**).

d-f The helix 44 and mtIF2 interactions. The helix 44 and mtIF2 are colored pink and blue, respectively. The cryo-EM densities are shown with the contour levels of 4.5, 4.5 and 5.0, respectively. In the mtIF2-mtIF3 bound complex, the density supports several interactions between mtIF2 and mtSSU through the helix 44 of 12S rRNA and uS1l (**e**). In the complete initiation complex, a poly alanine is placed in the mtIF2 region and no interaction from mtIF2 is observed (**f**), probably due to the conformational difference of the helix 44 of 12S rRNA.

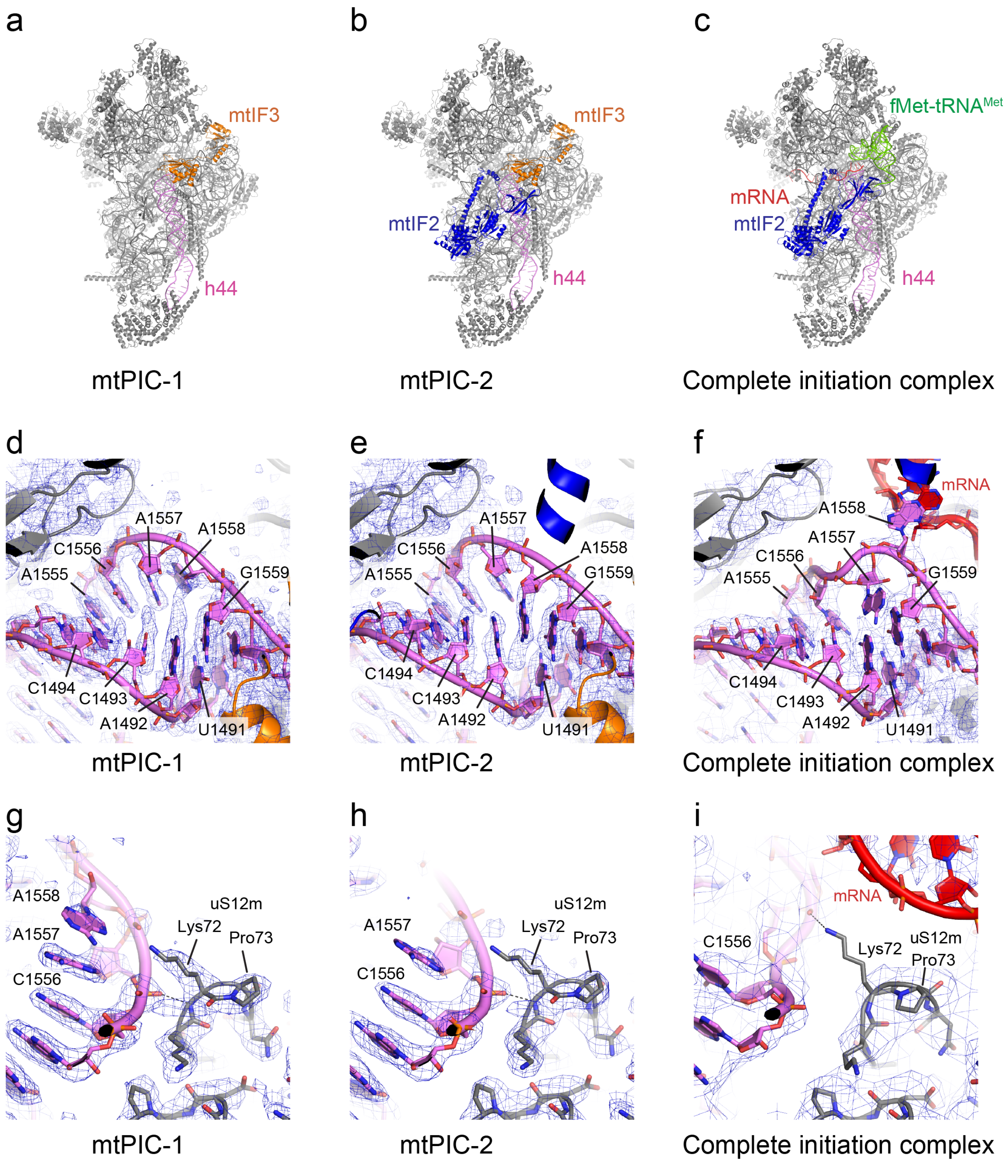

Supplementary Figure 14. Structure comparison of the helix 44 of 12S rRNA among the mtPICs and the complete initiation complex.

a-c Overall structures of mtPIC-1 (**a**), mtPIC-2 (**b**), and the complete initiation complex (**c**, PDB 6GAZ [3]). The helix 44, mtIF2, mtIF3, mRNA, and fMet-tRNA^{Met} are colored pink, orange, blue, red, and green, respectively. The helix 44 is surrounded by the factors. **d-f** Zoom-in views of the region 1492–1494 and 1557–1559 in the helix 44 from mtPIC-1 (**d**), mtI-2 (**e**), and the

corresponding region from the complete initiation complex (**f**). The cryo-EM densities are shown with the contour levels of 7.0, 7.0, and 6.0 (EMD-4369 [3]), respectively. Although the density is a mixture of a few alternative conformations in mtPIC-1 and mtPIC-2, the majority is different. In mtPIC-1, there is a gap of base stacking between C1493 and C1494 (**d**) and A1558 is stacked on A1557. On the other hand, in mtPIC-2, A1492 and A1558 occupy the positions of C1493 and A1492 in mtPIC-1, respectively, which makes continuous base stacking from C1491:G1559 pair, A1558, A1492, C1493, to C1495:G1554 pair (**e**). The two universally conserved adenosines, A1557 and A1558, are known to flip out from the helix when the A-site tRNA comes. The first adenosine flips out and is stacking on a mRNA base in the complete initiation complex (**f**). The residues of the porcine mt-ribosome in the complete initiation complex are labeled according to the residue numbering in human. **g-i** Interaction between the helix 44 and uS12m. The cryo-EM densities are shown with the contour levels of 8.0, 8.0, and 3.0, respectively. In mtPIC-1 (**g**) and mtPIC-2 (**h**), Lys72-Pro73 in uS12m forms a cis peptide so that the main-chain NH group of Lys72 interacts to the phosphate group of A1557 of the helix 44. In contrast, the interaction is missing in the complete initiation complex (**i**) due to the conformational difference of the helix 44, and the Lys72-Pro73 forming a trans peptide.

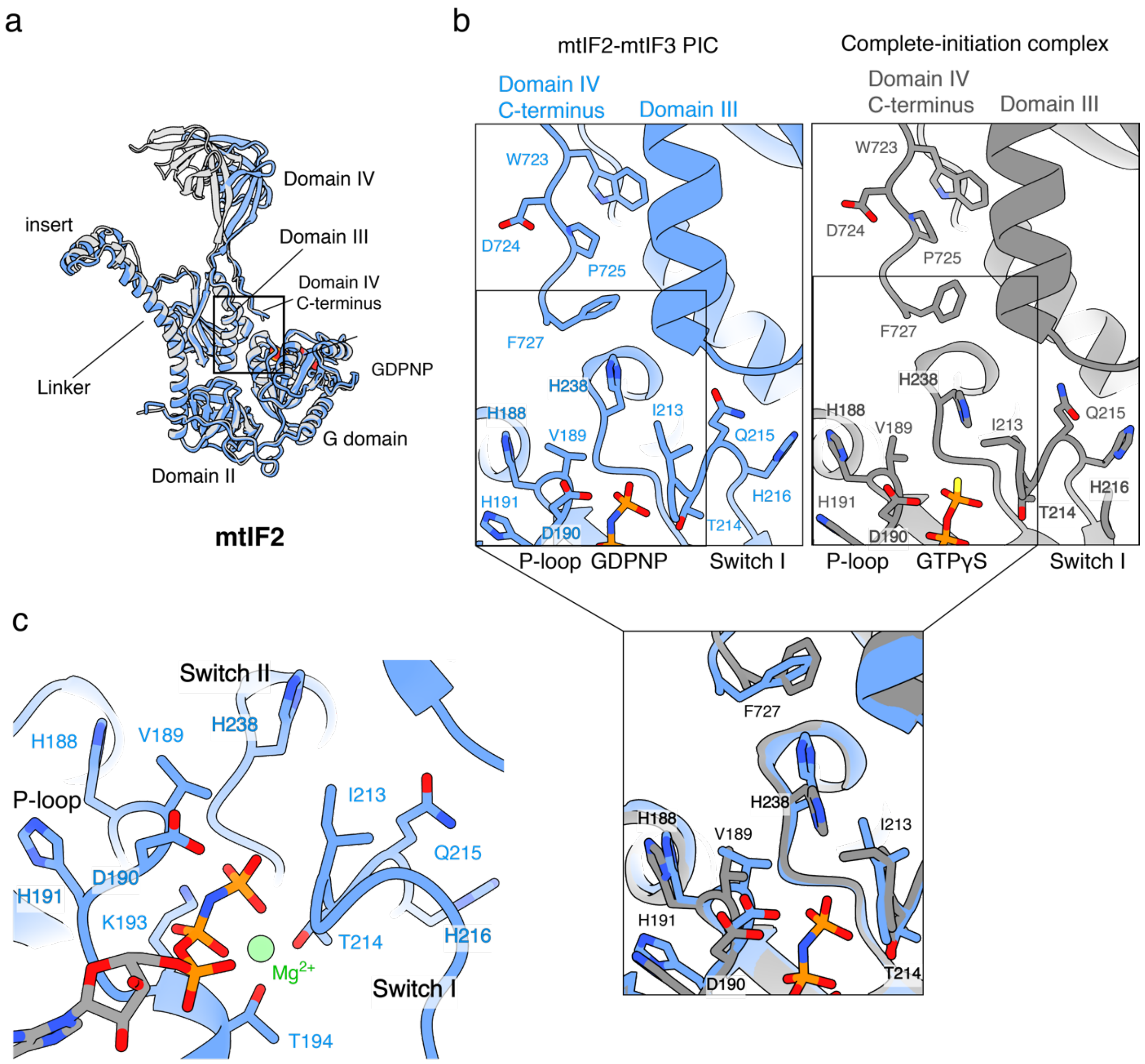

Supplementary Figure 15. mtIF2-dependent GTP hydrolysis.
a mtIF2 (blue) from the mtPIC-2 is superposed with mtIF2 (gray) from the complete initiation complex (PDB; 6GAW), shown independently of the mitoribosome. The C-terminus of domain IV and domain III lie in close proximity to the G-domain-bound GDPNP (boxed). **b** Zoom-in panels display the structures of the C-terminus of mtIF2 domain IV and the GTPase activation region from the mtPIC (blue) and the complete initiation complex (gray). Boxed areas from the above panels are superposed showing different conformational states for H238, V189, I213. **c** The mtIF2 G-domain bound-GDPNP shows several interactions with the P-loop, switch I and switch II. A magnesium ion (green, sphere representation) coordinates the β and γ phosphates of GDPNP. The conserved T214 (switch I) makes multiple interactions with the γ phosphate of GDPNP. The G4 (residues 288–291) and G5 (residues 324–326) motives interact with the guanosine moiety and the P-loop (residues 187–194) surrounds the phosphate groups of GDPNP.

Supplementary Table 1. Cryo-EM data collection, processing, model refinement, and validation statistics.

**Data collection**	mtPIC-1	mtPIC-2
Microscope	Titan Krios	Titan Krios
Detector	K2 Summit	K2 Summit
Magnification	165,000	165,000
Voltage [kV]	300	300
Total electron dose [e^-/Å^2]	30	30
Defocus range [µm]	−0.25 to −5.0	−0.25 to −5.0
Pixel size [Å]	0.83	0.83
Final particles	379,761	103,165
Resolution [Å] (Overall/ masked body core/ head core/ tail/ mS39 region/ mtIF bound region)	2.97/ 2.87/ 2.85/ 3.11/ 3.04/ 2.85	3.14/ 3.04// 3.02/ 3.38/ 3.38/ 3.00
Map-sharpening *B* factor [Å^2] (Overall/ masked body core/ head core/ tail/ mS39 region/ mtIF bound region)	−90/ −86/ −88/ −121/ −122/ −104	−84/ −83/ −89/ −122/ −116/ −73
**Model composition**		
Total atoms (non-hydrogen/ hydrogen)	68,869/ 59,430	73,335/ 63,948
Chains (RNA/ protein)	1/ 31	1/ 32
RNA residues (non-modified/ m^{5}U/ m^{4}C/ m^{5}C/ m$^6{}_2$A)	949/ 1/ 1/ 1/ 1/ 2	949/ 1/ 1/ 1/ 1/ 2
Protein residues (non-modified/ *N*-acetyl Ala)	5,918/ 2	6,489/ 2
Metal ions (Mg^{2+}/ K^+/ Zn^{2+})	60/ 17/ 1	61/ 17/ 1
Ligands (GDPNP/ ATP/ NAD^+/ 2Fe-2S/ spermine/ streptomycin)	1/ 1/ 1/ 2/ 1/ 1	2/ 1/ 1/ 2/ 1/ 1
Waters	59	61
**Refinement**		
Model to map CC (CC_{mask}/ CC_{box}/ CC_{peaks}/ CC_{volume})	0.84/ 0.85/ 0.80/ 0.84	0.85/ 0.86/ 0.80/ 0.85
Average *B* factor [Å^2] (Overall/ RNA/ protein/ metal ion and ligand/ water)	36.4/ 35.2/ 36.8/ 31.1/ 25.5	42.4/ 29.4/ 46.3/ 33.7/ 19.2
RMSD bond lengths [Å]	0.005	0.005
RMSD bond angles [°]	0.67	0.66
**Validation by MolProbity**		
Clash score	2.1	2.2
Rotamer outliers [%]	0.06	0.05

Ramachandran plot [%] (Favored/ allowed/ disallowed)	96.97/ 2.97/ 0.05	96.95/ 3.01/ 0.05
**EMDB ID** (Overall/ masked body core/ head core/ tail/ mS39 region/ mtIF bound region)	10021/ 10023/ 10024/ 10025/ 10026/ 10027	10022/ 10028/ 10029/ 10030/ 10031/ 10032/
**PDB ID**	6RW4	6RW5

Supplementary Table 2. Modeled RNA and proteins of the mtPIC.

Name	Uniprot ID	Chain ID	Modeled residues	Predicted mature RNA/protein	Notes
12S rRNA	NR_137294.1 (Ref_seq) NC_012920.1 (Ref_seq mt-gDNA)	A	648–1602	648–1601	A750G and A1438G variant.<br>Known modifications: m^5U1076, m^4C1486, m^5C1488, m^6_2A1583, m^6_2A1584.<br>NAD^+ interacting U948 and A1046.<br>Spermine interacting U944, G945, U946, U1044, G1045, C1048.<br>Streptomycin interacting G899, A1167, A1555, C1556.
bS1m	Q9Y2Q9	W	76–175	72–187	
uS2m	Q9Y399	B	53–277	1–296	Asp224, Asp240, Asp241 and His93 seem to coordinate an Mg^{2+} or K^+ ion instead of a Zn^{2+}.
uS3m	Q96EL2	C	36–167	36–167	
uS5m	P82675	D	88–430	1–430	
bS6m	P82932	E	2–123	1–125	Cys105 participates in a 2Fe-2S cluster coordination with bS18m.
uS7m	Q9Y2R9	F	35–242	38–242	
uS9m	P82933	G	50–176, 194–396	1–396	His51 seems interacting to an Mg^{2+} ion coordinated by 12S rRNA in the SSU body.
uS10m	P82664	H	50–189	1–201	
uS11m	P82912	I	58–194	1–194	
uS12m	O15235	J	31–138	30–138	Cis peptide at Pro73.
uS14m	O60783	K	28–128	1–128	
uS15m	P82914	L	63–236	58–257	
bS16m	Q9Y3D3	M	10–128	35–137	Cys26 participates in a 2Fe-2S cluster coordination with mS25.
uS17m	Q9Y2R5	N	4–113	21–130	
bS18m (bS18c)	Q9Y3D5	P	46–142	1–142	Cys65, Cys68 and Cys100 coordinate a 2Fe-2S cluster with bS6m Cys105.
bS21m	P82921	Q	2–87	1–87	N-acetylated Ala2. Cys50Arg variant.
mS22	P82650	R	64–358	1–360	
mS23	Q9Y3D9	S	2–136	2–190	
mS25	P82663	T	2–169	1–173	Cys139, Cys141 and Cys149 coordinate a 2Fe-2S cluster with bS16m Cys26.
mS26	Q9BYN8	U	27–202	28–205	
mS27	Q92552	V	29–293, 311–407	37–414	
mS29	P51398	X	47–398	22–398	GDP in the previous reports seems to be ATP with an Mg^{2+} ion.<br>Another nucleotide binds next to Tyr173, which is GTP or GDPNP.<br>Cis peptide at Pro339.
mS31	Q92665	Y	247–395	66–395	Cis peptide at Pro314.
mS33	Q9Y291	Z	3–102	2–106	
mS34	P82930	0	4–218	1–218	
mS35	P82673	1	48–323	?–323	
mS37	Q96BP2	2	2–118	1–118	N-acetylated Ala2.<br>Possible disulfide bonds of Cys45-Cys76 and Cys55-Cys66.
mS38	Q9NWT8	3	128–197	1–199	

mS39	Q96EY7	4	55–207, 232–666	38–689	Cis peptides at Pro135 and Pro483.
mS40 (bS18b)	Q9Y676	O	46–239	36–258	Zn^{2+} coordination (Cys94, Cys105, Cys108, Cys143).
mtIF2	P46199	7	157–727	30–727	GDPNP with an Mg^{2+} ion bound.
mtIF3	Q9H2K0	8	72–262	32–278	The68Ile, Phe243Leu variant.

Supplementary Table 3. Comparison of polar and stacking interactions between mtIF3 and mtSSU in this study and Koripella *et al.* [6].

mtIF3	Region	mtSSU	Type	Koripella et al.
Ser76 side chain	NTD	rRNA G988 phosphate	H bond	Not observed
Asn77 side chain	NTD	uS11m Thr114 main chain	H bond	Not observed
Asn77 main chain	NTD	uS11m Arg118 side chain	H bond	Not observed
Asp113 side chain	NTD	uS11m Arg138 side chain	H bond/ionic	Not observed
Arg115 side chain	NTD	rRNA A987 phosphate	H bond/ionic	Not observed
Arg115 side chain	NTD	uS11m Thr114 main chain	H bond	Observed
Gly133 main chain	Linker	rRNA A987 ribose	H bond	Not observed
Leu137 side chain	Linker	rRNA A987 base	Stacking	Observed
Arg140 side chain	Linker	rRNA A987 base	H bond	Not observed
Arg140 side chain	Linker	rRNA A987 ribose	H bond	Observed
Arg140 side chain	Linker	rRNA G988 base	Stacking	Observed
Arg140 side chain	Linker	rRNA U1000 base	H bond	Not observed
Arg144 side chain	Linker	rRNA C999 phosphate	H bond/ionic	Observed
Arg144 side chain	Linker	rRNA U1000 phosphate	H bond/ionic	Observed
Lys159 side chain	CTD	rRNA U1077 base	H bond	Not observed
Lys159 side chain	CTD	rRNA A1080 phosphate	H bond/ionic	Not observed
Glu160 main chain	CTD	rRNA A1078 ribose	H bond	Observed
Glu160 main chain	CTD	rRNA A1079 phosphate	H bond	Not observed
Ser164 side chain	CTD	rRNA C1561 phosphate	H bond	Not observed
Asn166 side chain	CTD	rRNA U1560 phosphate	H bond	Not observed
Gly168 main chain	CTD	rRNA U1560 ribose	H bond	Observed
His170 side chain	CTD	rRNA U1490 ribose	H bond	Not observed
Asp171 side chain	CTD	rRNA U1560 ribose	H bond	Observed
Lys175 side chain	CTD	rRNA G1562 phosphate	H bond/ionic	Observed
Gln178 side chain	CTD	rRNA G1079 ribose	H bond	Observed but differently

Trp182 side chain	CTD	rRNA A1080 phosphate	H bond	Not observed

All the listed interactions are supported by the cryo-EM density of mtPIC-1.
Simple Van der Waals (VDW) interactions are not listed.
All the interactions described in Koripella et al. [6] are observed, except for those through mtIF3 Phe75, Val78, Arg80 and Lys81. Phe75 is away from mtSSU in our model. Val78 is away from rRNA, rather than in VDW distance from Gly116 of uS11m. Arg80 side chain does not point to mtSSU, rather than making an internal salt bridging with Asp110. Lys81 in our model seems to point to uS7m in the head of mtSSU, instead to uS11m.
The differences seem to be due to the resolution improvement.

Supplementary Table 4. *In vitro* translation initiation assay using fluorescently labeled components: Cy5-mtIF3, Cy3-fMet-tRNA$^{Met}_{i}$ and Atto390-mRNA.

	**1**	**2**	**3**	**4**	**5**	**6**
	**mtSSU+ mtIF2 + mtIF3**	**mtSSU + mtIF2 + tRNA**	**mtSSU+ mtIF2 + mtIF3 + mRNA**	**mtSSU+ mtIF2 + tRNA + mRNA**	**mtSSU + mtIF2 + mtIF3 + tRNA**	**mtSSU+ mtIF2 + mtIF3 + tRNA + mRNA**
**mtIF3-Cy5**	58±7%	N/A	55±8%	N/A	34±5%	41±7%
**fMet-tRNA-Cy3**	N/A	23±6%	N/A	26±2%	22±3%	20±4%
**mRNA-Atto390**	N/A	N/A	0	0	N/A	0

Translation initiation reactions were performed as described in methods, using the fluorescently labeled components presented in the Table. After purification of the initiation complex, the concentration of each labeled species was measured and normalized to the mtSSU concentration. We did not detect fluorescence of the Atto390-labeled mRNA in any of the described conditions, indicating that the mRNA is not loaded on the mtSSU, but only after binding of the mtLSU. The data presented in the Table were complemented with the fluorescence cross-correlation spectroscopy analysis (Fig. 3b and Supplementary Fig. 9), to investigate if mtIF3 and tRNA bind to the same mtSSU. Data are derived from the mean values (n=3). Error bars represent the standard deviation. Source data are provided as a Source Data file.

Supplementary Table 5. Polar and stacking interactions between mtIF2 and mtSSU in the mtPIC-2 and the complete initiation complex.

mtIF2	Region	mtSSU	Type	Kummer et al. [3]
Lys366 main chain	Linker	rRNA U821 ribose	H bond	Observed
Gly367 main chain	Linker	rRNA A695 base	Stacking	Observed
Lys383 main chain	Linker	rRNA U832 phosphate	H bond/ionic	Not observed
Arg397 side chain	Linker	rRNA U821 phosphate	H bond/ionic	Observed
Arg397 side chain	Linker	rRNA U831 phosphate	H bond/ionic	Observed
Asp459 side chain	Linker	uS12m Thr101 side chain	H bond	Observed
Lys466 side chain	Insertion	uS12m His100 main chain	H bond	Not observed
Arg467 main chain	Insertion	rRNA A892 ribose	H bond	Observed
His470 side chain	Insertion	rRNA C891 ribose	H bond	Observed
His474 side chain	Insertion	rRNA C890 phosphate	H bond	Observed
Gln475 side chain	Insertion	rRNA G889 phosphate	H bond	Observed
Arg478 side chain	Insertion	rRNA G889 ribose	H bond/ionic	Observed
Arg478 side chain	Insertion	rRNA C890 phosphate	Ionic	Observed
Trp486 side chain	Insertion	rRNA G902 base	Stacking	Observed
Lys504 main chain	Linker	rRNA C1493 ribose	H bond	Not observed
Lys504 side chain	Linker	rRNA C1494 base	H bond	Not observed
Lys506 side chain	Linker	rRNA C1495 phosphate	H bond/ionic	Not observed
Glu507side chain	Linker	uS12m Phe66 side chain	Stacking	Not observed
Glu507 side chain	Linker	uS12m Arg68 side chain	H bond/ionic	Not observed
Lys508 side chain	Linker	rRNA U1496 phosphate	H bond/ionic	Not observed
Arg509 main chain	Linker	uS12m Cys64 side chain	H bond	Not observed

All the listed interactions are supported by the cryo-EM density of mtPIC-2.
Simple VDW interactions are not listed.
All the interactions described in Kummer et al. [3] are observed, except for those through mtIF2 Lys487, Arg489, Ser490, and Phe949.
The interactions through the loop consists of the residue 479–513 in mtIF2 are different due to the conformational difference of h44 of rRNA.
