# Supplementary material for: Distinct pre-initiation steps in human mitochondrial translation
Source: Nat Commun. 2020 Jun 10;11:2932. doi: 10.1038/s41467-020-16503-2 (PMC7287080; doi:10.1038/s41467-020-16503-2)
Supplement: Supplementary file 3 — Reporting Summary [file 41467_2020_16503_MOESM3_ESM.pdf]

nature research

Corresponding author(s): Joanna Rorbach, Alexey Amunts
Last updated by author(s): 30/3/20

Reporting Summary

Nature Research wishes to improve the reproducibility of the work that we publish. This form provides structure for consistency and transparency in reporting. For further information on Nature Research policies, see Authors & Referees and the Editorial Policy Checklist.

Statistics

For all statistical analyses, confirm that the following items are present in the figure legend, table legend, main text, or Methods section.

n/a	Confirmed	
☐	☒	The exact sample size (*n*) for each experimental group/condition, given as a discrete number and unit of measurement
☐	☒	A statement on whether measurements were taken from distinct samples or whether the same sample was measured repeatedly
☐	☒	The statistical test(s) used AND whether they are one- or two-sided<br>*Only common tests should be described solely by name; describe more complex techniques in the Methods section.*
☒	☐	A description of all covariates tested
☒	☐	A description of any assumptions or corrections, such as tests of normality and adjustment for multiple comparisons
☐	☒	A full description of the statistical parameters including central tendency (e.g. means) or other basic estimates (e.g. regression coefficient) AND variation (e.g. standard deviation) or associated estimates of uncertainty (e.g. confidence intervals)
☒	☐	For null hypothesis testing, the test statistic (e.g. *F*, *t*, *r*) with confidence intervals, effect sizes, degrees of freedom and *P* value noted<br>*Give P values as exact values whenever suitable.*
☒	☐	For Bayesian analysis, information on the choice of priors and Markov chain Monte Carlo settings
☒	☐	For hierarchical and complex designs, identification of the appropriate level for tests and full reporting of outcomes
☒	☐	Estimates of effect sizes (e.g. Cohen's *d*, Pearson's *r*), indicating how they were calculated

Our web collection on statistics for biologists contains articles on many of the points above.

Software and code

Policy information about availability of computer code

Data collection	Images were collected on a Titan Krios electron microscope (Thermofisher) operated at 300 kV and equipped with a K2 Summit direct electron detector (Gatan). Micrographs were obtained from automated data collections (EPU software, Thermofisher) at 165,000× magnification, yielding a pixel size of 0.83 Å. 4 sec exposures yielded a total dose of 30 e-/Å2 in 20 frames, with defocus values ranging from -0.2 to -5.0 µm. A total of 3,335 micrographs were recorded and kept.
Data analysis	Movie frames were aligned and averaged by global and local motion corrections by the program MotionCor2. Contrast transfer function (CTF) parameters were estimated by GCTF. Particles were picked by Gautomatch and 2D classified by RELION 3.<br>The models were manually built with Coot and stereochemical refinement was performed using phenix.real_space_refine in the PHENIX suite. The final model was validated using MolProbity.

For manuscripts utilizing custom algorithms or software that are central to the research but not yet described in published literature, software must be made available to editors/reviewers. We strongly encourage code deposition in a community repository (e.g. GitHub). See the Nature Research guidelines for submitting code & software for further information.

Data

Policy information about availability of data

All manuscripts must include a data availability statement. This statement should provide the following information, where applicable:

- Accession codes, unique identifiers, or web links for publicly available datasets
- A list of figures that have associated raw data
- A description of any restrictions on data availability

The coordinates and corresponding cryo-EM maps were deposited in the Protein Data Bank (PDB) and in the Electron Microscopy Data Bank (EMDB) under accession codes: 6RW4 and 10021/ 10023/ 10024/ 10025/ 10026/ 10027 for mtPIC-1 and 6RW5 and 10022/ 10028/ 10029/ 10030/ 10031/ 10032 for mtPIC-2.

Field-specific reporting

Please select the one below that is the best fit for your research. If you are not sure, read the appropriate sections before making your selection.

☒ Life sciences ☐ Behavioural & social sciences ☐ Ecological, evolutionary & environmental sciences

For a reference copy of the document with all sections, see nature.com/documents/nr-reporting-summary-flat.pdf

Life sciences study design

All studies must disclose on these points even when the disclosure is negative.

Sample size	At least 3 replicates have been included.
Data exclusions	No data was excluded.
Replication	For our in vitro biochemical experiments all attempts at replication were successful.
Randomization	Not relevant to our study.
Blinding	Not relevant to our study.

Reporting for specific materials, systems and methods

We require information from authors about some types of materials, experimental systems and methods used in many studies. Here, indicate whether each material, system or method listed is relevant to your study. If you are not sure if a list item applies to your research, read the appropriate section before selecting a response.

Materials & experimental systems

n/a	Involved in the study	
☒	☐	Antibodies
☐	☒	Eukaryotic cell lines
☒	☐	Palaeontology
☒	☐	Animals and other organisms
☒	☐	Human research participants
☒	☐	Clinical data

Methods

n/a	Involved in the study	
☒	☐	ChIP-seq
☒	☐	Flow cytometry
☒	☐	MRI-based neuroimaging

Eukaryotic cell lines

Policy information about cell lines

Cell line source(s)	Flp-In Trex 293 cell line was purchased from Invitrogen
Authentication	Flp-In Trex 293 cells were authenticated by having resistance to Blasticidin and Zeocin.
Mycoplasma contamination	Cell line tested negative for mycoplasma contamination
Commonly misidentified lines (See ICLAC register)	N/A
