# Supplementary material for: Distinct pre-initiation steps in human mitochondrial translation
Source: Nat Commun. 2020 Jun 10;11:2932. doi: 10.1038/s41467-020-16503-2 (PMC7287080; doi:10.1038/s41467-020-16503-2)
Supplement: Supplementary file 2 — Peer Review File [file 41467_2020_16503_MOESM2_ESM.pdf]

Editorial Note: This manuscript has been previously reviewed at another journal that is not operating a transparent peer review scheme. This document only contains reviewer comments and rebuttal letters for versions considered at Nature Communications.

REVIEWERS' COMMENTS:

Reviewer #1 (Remarks to the Author):

In my original review, I had concerns that mtIF3 might move on the mtSSU to accommodate tRNA. The additional structural analysis provided in the revised manuscript has allayed my concerns.

My other major concern was that insufficient evidence was provided in the original manuscript to support the model that mRNA binds to the monosome but not the SSU. Here, the authors provide further support for their "monosome only" model through the use of single-molecule imaging (optical tweezers combined with confocal fluorescence microscopy). These experiments are a great addition to the paper.

In light of the improvements to the manuscript, I recommend that the paper is published. The findings are novel and the structural biology is done to a very high standard.

Reviewer #2 (Remarks to the Author):

The initiation of mitochondrial translation is a process of fundamental importance, which is still poorly characterized. Here Khawaja et al. have studied the initiation of translation in mitochondria. A biochemical reconstitution included a mitochondrial small subunit (mtSSU) bound to mtIF3 to which recombinant mtIF2-GMPPNP and initiator tRNA were added. Samples of this reaction mixture were subsequently analyzed by cryo-EM to reveal two distinct states. The first state contains mtSSU-mtIF3 whereas the second contains mtSSU-mtIF2-mtIF3. The structural biology analysis that is presented in this manuscript is of exceptional quality and the interpretation of the structural data is outstanding.

Subsequent biochemical studies (using fluorescence cross-correlation spectroscopy and single-molecule imaging) were used to obtain further insights into the mechanism of mitochondrial translation initiation where the authors found that monosomes but not mtSSU complexes are associated with messenger RNAs. The authors conclude that there are key differences between bacterial and mitochondrial translation initiation so that during mitochondrial translation initiation mRNA only associates with monosomes and not mtSSU complexes.

The authors have revised several key points after their initial submission, and I believe that this manuscript is of clear interest to the readership of Nature Communications and should therefore be published provided that the following point is addressed:

The available biochemical data can be explained by two different models for mitochondrial translation initiation (1. mRNA associates late (with monosomes; c.f. Figure 5) or 2. mRNA associates very weakly with mtSSU complexes earlier) while the authors have only illustrated the first model in Figure 5.

The existing biochemical data shows that:

1. Monosomes are the only species that is found to be stably associated with mRNAs when using FCCS (Fig.2).
2. Monosomes are the only species that stably bind to mRNA in single-molecule experiments (Fig. 5).

The existing biochemical data however does not rule out that:

1. mtSSU-mRNA complexes are very transient and cannot be detected with the employed techniques.

In the absence of Shine-Dalgarno and anti-Shine-Dalgarno sequences it is expected that mtSSU-mRNA interactions are weaker than in bacteria. The addition of the mtLSU would be expected to enhance mRNA affinity so that monosomes should have a higher affinity for mRNAs than mtSSUs. This is consistent with the data shown in Figures 2 and 5.

Based on the above points, it is equally possible (but only stated by the authors in the last two sentences of the manuscript) that mitochondrial translation initiation could involve mRNA joining at an earlier point via transient intermediates.

To address this point clearly, the easiest way would be to change the model proposed in Figure 5 so that mRNA joins at the same time as mtLSU and initiator tRNA because the current temporal resolution does not allow us to distinguish between these options. Importantly, this model would:

a. Capture all available data as this point is still not fully addressed.
b. Include a model in which mRNA may bind after mtLSU or before mtLSU binding.
c. Leave enough room for further in-depth mechanistic analyses, which are beyond the scope of this manuscript, to be performed in the future.

Reviewer #3 (Remarks to the Author):

I had the pleasure of reviewing this manuscript in its previous incarnation. I was thoroughly impressed and found it compelling, as it reports a translation initiation pathway that is unique to mitochondria, albeit that it may occasionally occur in bacteria. The authors have now performed some very delicate molecular tweezers expts to address comments from a reviewer who has suggested that weak interaction of various components in the pre-initiation pathway may have been lost during sucrose gradient centrifugation. This new data fully supports the authors pathway. I very much like this work and strongly support its publication. In the Life Sciences, nothing is ever fully black and whilte. Of course, there are some highly unlikely scenarios that may occur that could argue against the data presented to support this pathway, but I think the authors have done more than enough to lead to publication of what is quite a provocative and exciting novelty that once again supports the idea that mitochondria do things their own way!

We thank the referees for their constructive inputs that helped us to improve the manuscript, and are happy that they are satisfied with our revision. The only outstanding point was raised by referee 2, and we fully addressed it as requested by changing the proposed Figure 5 so that mRNA joins at the same time as mtLSU. More detailed response is described below:

Referee 2:
"The existing biochemical data however does not rule out that:

1. mtSSU-mRNA complexes are very transient and cannot be detected with the employed techniques.

In the absence of Shine-Dalgarno and anti-Shine-Dalgarno sequences it is expected that mtSSU-mRNA interactions are weaker than in bacteria. The addition of the mtLSU would be expected to enhance mRNA affinity so that monosomes should have a higher affinity for mRNAs than mtSSUs. This is consistent with the data shown in Figures 2 and 5.

Based on the above points, it is equally possible (but only stated by the authors in the last two sentences of the manuscript) that mitochondrial translation initiation could involve mRNA joining at an earlier point via transient intermediates.

To address this point clearly, the easiest way would be to change the model proposed in Figure 5 so that mRNA joins at the same time as mtLSU and initiator tRNA because the current temporal resolution does not allow us to distinguish between these options. Importantly, this model would:

a. Capture all available data as this point is still not fully addressed.
b. Include a model in which mRNA may bind after mtLSU or before mtLSU binding.
c. Leave enough room for further in-depth mechanistic analyses, which are beyond the scope of this manuscript, to be performed in the future."

We agree with the referee that with our current experimental setup we are not able to discriminate whether mRNA binds before or after mtLSU joining.
We have now modified the model in Figure 7 (previously Figure 5) to accommodate both possibilities. We also highlighted it in the figure legend:
'Joining of the mtLSU may result in the conformational change of mtIF2 and GTPase activation that leads to tRNA and mRNA accommodation. Alternatively, transient binding of mRNA and tRNA (not detected with our techniques) to the mtSSU after mtIF3 departure precede recruitment of the mtLSU.'

Finally, in the abstract 'reveal' was replaced with 'suggest' in order not to rule out other possible states.
